# Atomic‐Scale Insights into the 2D Materials from Aberration‐Corrected Scanning Transmission Electron Microscopy: Progress and Future

**DOI:** 10.1002/smsc.202300073

**Published:** 2023-12-14

**Authors:** Woonbae Sohn, Miyoung Kim, Ho Won Jang

**Affiliations:** ^1^ Department of Materials Science and Engineering Research Institute of Advanced Materials Seoul National University Seoul 08826 Republic of Korea; ^2^ Advanced Institute of Convergence Technology Seoul National University Suwon 16229 Republic of Korea

**Keywords:** 2D materials, crystal structures, defects, heterostructures, scanning transmission electron microscopy

## Abstract

2D crystals are attractive due to their unique atomic, electronic structures, and physiochemical properties, which strongly rely on the synthesis conditions. The atomic structure and presence of defects in the crystal lattice, such as vacancies, dopants, grain boundaries, and edge terminations, significantly influence the properties of 2D materials. Due to its high spatial resolution, aberration‐corrected scanning transmission electron microscopy (AC‐STEM) has become a powerful tool to provide atomic‐scale insights into the crystal structure, defects, heterointerfaces, ferroelectricity, and in situ observations of 2D materials. This review will cover the status of atomic‐scale studies on various 2D materials, including graphene, boron nitride, transition metal dichaogenides, MXenes, and phosphorene using AC‐STEM. The future perspective of AC‐STEM for new findings in 2D materials using machine learning is further discussed.

## Graphical abstract

The status of atomic‐scale studies on various 2D materials such as graphene, boron nitride, transition metal dichaogenides, MXenes, and phosphorene using aberration‐corrected scanning transmission electron microscopy (AC‐STEM) is reviewed. Future perspectives for AC‐STEM in new findings of 2D materials using in situ observations and machine learning are further discussed.

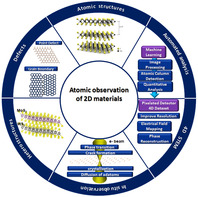

## 1 Introduction

2D materials refer to a class of materials that possess extraordinary properties and consist of a single layer or a few layers of atoms arranged in a 2D plane. These materials exhibit unique characteristics and behaviors that arise from their reduced dimensionality, such as exceptional mechanical strength, high electrical conductivity, and remarkable optical properties. The most well‐known example of a 2D material is graphene, which is composed of a single layer of carbon atoms arranged in a hexagonal lattice. Other examples include transition metal dichalcogenides (TMDs) like molybdenum disulfide (MoS_2_) and hexagonal boron nitride (h‐BN). The field of 2D materials has attracted significant research interest due to their potential applications in electronics, photonics, energy storage, and various other technological advancements.

2D materials with ultrathin thickness, which have witnessed a significant expansion since the first isolation of graphene was reported,^[^ ^1^ ^]^ present themselves as potential candidates for next‐generation electronics and optoelectronics, thanks to their unique features.^[^ ^2^, ^3^, ^4^, ^5^ ^]^ There are several kinds of 2D materials which include graphene, transition metal chalcogenides, MXenes, and phosphorene.

Understanding the atomic structure (atomic configuration) of 2D materials is crucial for clarifying their atomic and crystal structures and establishing the structure–property relationship. Even a point defect or the number of layers can strongly influence their electromagnetic and optical properties. Several powerful methods, including aberration‐corrected scanning transmission electron microscopy (AC‐STEM), have been employed for direct observation of the atomic structure in 2D materials, complementing techniques such as atomic force microscopy (AFM), scanning tunneling microscopy (STM), and X‐ray photoemission spectroscopy (XPS). These techniques collectively provide a comprehensive understanding of the structural properties of 2D materials.

AC‐STEM, with its subangstrom resolution,^[^ ^6^, ^7^, ^8^, ^9^, ^10^, ^11^, ^12^, ^13^ ^]^ enables the precise identification of atomic columns in 2D materials. It can also detect defects, dopants, and other structural features, offering valuable insights into the atomic arrangement. To establish a complete structure–property relationship, a combination of AC‐STEM observation, density functional theory (DFT) calculations, XPS, and AFM can be utilized in material science studies. These techniques collectively provide a comprehensive understanding of the structural and electronic properties of 2D materials.

However, the ultrathin nature of 2D materials makes them sensitive, highly reactive, and vulnerable to electron beam‐induced damage. Consequently, AC‐STEM imaging must be carried out at low accelerating voltages (60–80 kV) to minimize beam damage. Higher accelerating voltages can induce reactions with the surface of the 2D materials, leading to atomic loss, chemical reactions, and compromising the pristine status of the material.^[^ ^14^, ^15^ ^]^ Therefore, careful consideration of the imaging conditions is essential when performing AC‐STEM on 2D materials.

In addition to AC‐STEM, other techniques such as AFM, STM, and XPS also contribute to the characterization of 2D materials. AFM and STM offer surface topography and electronic information at the atomic scale, respectively. XPS provides chemical composition and electronic state information. AFM is limited to surface characterization and does not provide direct atomic‐scale information. In case of STM, only conductive materials and surfaces can be detected. In case of XPS, spatial resolution is lacking. Likewise, AC‐STEM, AFM, STM, and XPS are complementary to each other. By combining these techniques with AC‐STEM, a comprehensive understanding of 2D materials can be achieved.

STEM with an aberration corrector provides atomic resolution for the visualization of 2D materials. An annular dark field STEM (ADF‐STEM) image is obtained by inserting an annular detector to collect scattered electrons. High‐angle ADF‐STEM (HAADF‐STEM) has high contrast which is proportional to *Z* ^*x*^ (*x* = 1.7–2.0) and is beneficial to detect heavy elements. Medium and low scattering angles, called MAADF and LAADF‐STEM imaging, can also be used for atomic‐resolution analysis of 2D materials, with less dependence on Z contrast.^[^ ^16^ ^]^

In this review, we introduce what atomic insights of 2D materials have been provided with the aid of AC‐STEM in five main sections: atomic structures, defects, heterostructures, in situ observation, and data‐driven and computational analysis.

## 2 Revealing Atomic Structures of 2D Materials

In this section, we will explore the atomic structures of various 2D materials, including graphene, TMDs (with formula *MX*_2_ where *M* = Mo, W and *X* = S, Se), BN, and MXenes. Additionally, we will discuss the polymorphism observed in TMDs.

### 2.1 Graphene

Graphene, which is composed of a single layer of atoms arranged in a 2D honeycomb lattice, has attracted attention as a material that can be observed using AC‐STEM. Observing the atomic arrangements and defects like vacancies, dislocations, and grain boundaries (GBs) in graphene is crucial for gaining insights into its mechanical, electromagnetic, and chemical properties. Nonetheless, the carbon–carbon bonds become susceptible to damage from electron beam irradiation at high voltages, rendering the observation of graphene unfeasible. Thus, AC‐STEM observation of graphene should be carried out under low voltage (under 100 kV) in the transmission electron microscope, leading to low signal–noise ratio.^[^ ^17^ ^]^ Thus, image processing such as summation of intensity, background subtraction, and filtering is necessary to get clear STEM image of hexagonal graphene. In 2011, Huang et al. first observed the atomic structure of single‐layer graphene with GBs using AC‐STEM.^[^ ^17^ ^]^ This study not only showed atomic images of graphene with dislocations and GBs but also suggested the optimal optical condition of visualization of the 2D materials. Further observations of graphene such as dopants, GBs, and dislocations are covered in the next section.

### 2.2 BN

BN is a compound composed of boron and nitrogen atoms arranged alternately, and it is known as the lightest Group III–V compound. The atomic structure of h‐BN sheets bears resemblance to graphene, but with a notable difference. Unlike graphene, h‐BN contains hetero atoms, namely, carbon and nitrogen. As a result, the electrons in h‐BN localize to the nitrogen sites, making it a dielectric and an insulator with a direct bandgap of approximately 5.9 eV. This characteristic makes h‐BN suitable for applications such as graphene‐based field‐effect transistors and dielectric gates.

In the initial stages of studying the 2D structure of h‐BN, research primarily focused on its topology, edge structures, and defects such as point defects, dislocations, and GBs.^[^ ^12^, ^13^, ^16^, ^18^, ^19^, ^20^ ^]^ Later, the atomic structure of h‐BN gained attention, specifically in relation to its stacking sequence.^[^ ^21^ ^]^ Subsequently, a combination of STEM studies and DFT calculations enabled the investigation of not only the atomic configuration of defects but also the electronic structures^[^ ^22^, ^23^, ^24^ ^]^ and the relationship between structure and properties^[^ ^25^ ^]^ of defect or substitutional sites in h‐BN.

More recently, the development of integrated differential phase contrast (iDPC)‐STEM has made it possible to map electric fields at the atomic level. Cretu et al. demonstrated the distribution of electric fields in h‐BN with defects. Their study revealed that the presence of monovacancies or extended defect edges in h‐BN leads to an increase in electric field compared to the nondefect crystalline sites.^[^ ^26^ ^]^

### 2.3 Polymorphs of TMDs

**Figure 1:**
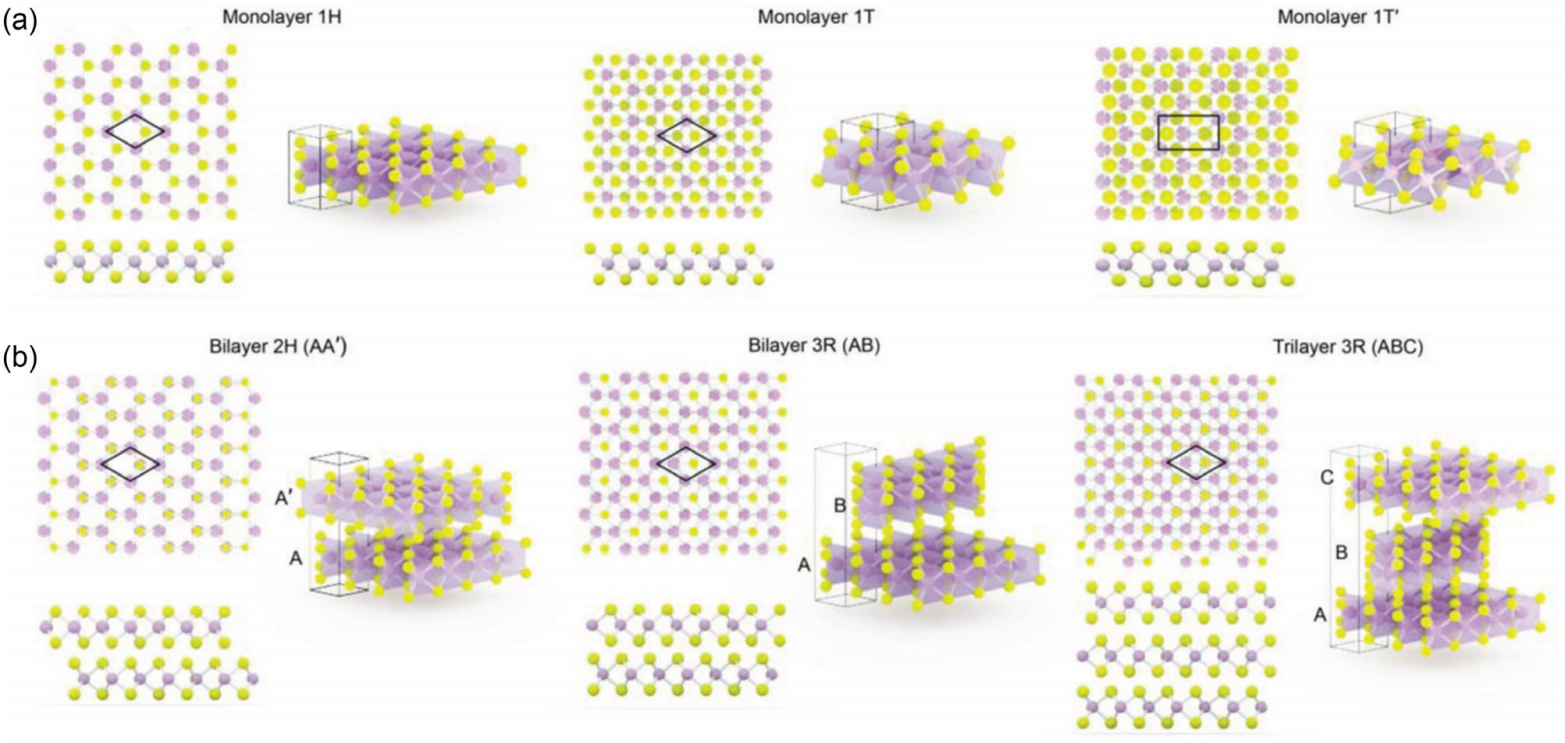
Atomic structures of various polymorphs in MoS_2_. a,b) Atomic models of monolayer 1H, 1T, 1T′ (a), and bilayer 2H, 3R, and trilayer 3R‐stacked MoS_2_ (b), respectively. The side views and perspective views are depicted in the lower and right panels, respectively. a,b) Adapted with permission.^[^ ^35^ ^]^ Copyright 2015, Wiley‐VCH.

TMD materials, especially MoS_2_, provide a wide range of polymorphs depending on layer stacking sequences. The most prevalent among them include semiconductor 1H‐MoS_2_,^[^ ^27^, ^28^, ^29^ ^]^ semimetal 1T′‐MoS_2_,^[^ ^30^, ^31^, ^32^ ^]^ and metallic 1T‐MoS_2_,^[^ ^33^, ^34^ ^]^ which shows that the phases depend on the stacking order of layers (see **Figure** **1**a). The layer‐by‐layer stacking sequence determines not only phases but also the band structures of MoS_2_, leading to a distinct electromagnetic property.^[^ ^35^, ^36^ ^]^

**Figure 2:**
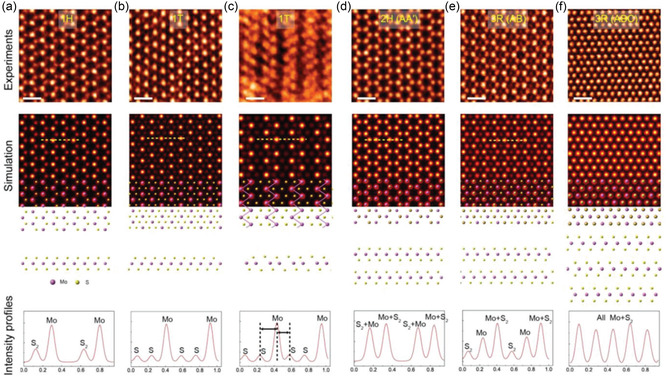
a–f) ADF‐STEM images of bilayer 1H (a), 1T (b), 1T′ (c), 2H (d), 3R (AB stacking) (e), and 3R (ABO stacking) (f) phase MoS_2_ followed by multislice image simulation, atomic model, and intensity profiles. a,b) Adapted under the terms of the CC‐BY Creative Commons Attribution 4.0 International license (https://creativecommons.org/licenses/by/4.0).^[^ ^42^ ^]^ Copyright 2016, The Authors, published by Wiley‐VCH. b) Adapted with permission.^[^ ^43^ ^]^ Copyright 2016, American Chemical Society. c) Adapted with permission.^[^ ^32^ ^]^ Copyright 2016, American Chemical Society (https://doi.org/10.1021/acsnano.6b05746; further permissions for the material excerpted should be directed to the ACS). d–f) Adapted with permission.^[^ ^40^ ^]^ Copyright 2018, Wiley‐VCH.

**Figure** **2** shows how the polymorphic phase of MoS_2_ can be distinguished by identifying intensity of Mo and S atomic columns. In case of the monolayer MoS_2_ in 1H and 1T phase (Figure 2a,b), S, Mo, S atomic layers are arranged alternatively.^[^ ^35^, ^36^ ^]^

The 1H and 1T crystal structures have different layer‐by‐layer stacking orders. Specifically, the stacking order for 1H is ABA, while for 1T it is ABC. 1T‐MoS_2_ is thermodynamically less stable than 1H one, and Peierls distortion happens to reduce the dimensionally of the 2D system into three equivalent 1D zigzag (ZZ) chains, giving rise to the so‐called 1T′ phase, as shown in Figure 2c.^[^ ^4^, ^6^ ^]^ In the case of multilayer H phase MoS_2_, several polymorphs of TMDs have been reported, including 2H and 3R, as shown in Figure 2d–f. These polymorphs have different stacking sequences, with 2H having an AA′ stacking and 3R having an AB or ABC stacking.^[^ ^35^, ^36^ ^]^

The intensity and position of sulfur atomic columns in the STEM‐ADF image can distinguish monolayer 1T‐ and 1H‐MoS_2_ intuitively. Figure 2a,b displays both phases’ experimental and simulated STEM‐ADF images, with marked atomic columns. The simulated intensity of the atomic column containing the S dimer in the 1H phase image is 2 times larger that in 1T phase image because of the overlap of the two sulfur atomic layers in the 1H phase along the [001] zone axis. Because of surface contamination and nonlinear relationship between the image intensity and number of overlapped atomic columns, resolving single sulfur atomic columns in the 1T phase is challenging.^[^ ^37^, ^38^ ^]^ To enhance the contrast and hence identify S atomic columns in 1T‐MoS_2_, it is necessary to observe MoS_2_ with a lower accelerating voltage and use of the medium‐angle annular dark field (MAADF) detector.

In the case of STEM imaging of multilayer films, however, the presence of “stacking polytypes” makes the figuring out polymorphs more complicated, confusing, and thus challenging. For example, the STEM image of bilayer AA‐stacked 1T film is same as that of bilayer AB′‐stacked 1H film.^[^ ^39^ ^]^ The STEM‐ADF image of the 1T′‐MoS_2_ phase presented in Figure 2c displays distinct Mo zigzag chains exhibiting periodic contraction and elongation of in‐plane S–S. This is clearly shown in the intensity line profile. While bilayer 2H‐MoS_2_ (Figure 2d) and monolayer 1H‐MoS_2_ (Figure 2a) have a similar projected 2D atomic arrangement, the image contrast of Mo and S_2_ atom columns in the honeycomb of 1H‐MoS_2_ (Figure 2a) is distinguishable, whereas the intensity variations of Mo + S_2_ and S_2_ + Mo atomic columns in 2H‐MoS_2_ (Figure 2d) are quite similar. Bilayer 3R‐stacked MoS_2_ (Figure 2e) can be identified from 2H by the faint areas present in the center of each hexagon.^[^ ^40^, ^41^, ^42^, ^43^ ^]^ Although the atomic configurations of 1T‐MoS_2_ and bilayer 3R‐MoS_2_ seem comparable, there is a distinguishable dissimilarity in the strength of the three‐column atom peaks in bilayer 3R‐MoS_2_. The contrast between the Mo + S_2_, Mo, and S_2_ atomic columns within each unit cell diminishes in bilayer 3R‐MoS_2_. In the 1T phase, the contrast between the two S monomers is equally weak and much weaker than the contrast of the Mo atom, which may cause them to blend into the background intensity (see Figure 2d,e). In trilayer 3R‐MoS_2_, all atom columns contain an equivalent number of atoms so that distinguishing contrast variations is nearly impossible (see Figure 2f).

### 2.4 Black Phosphorous

Black phosphorous (BP) is a kind of allotrope of phosphorous. Its bandgap depends on the number of layers (2 eV from monolayer to 0.3 eV from bulk). BP forms a 2D‐layered structure just like graphene. However, the major difference from the graphene is that in case of BP, there are two distinguishable directions at the edge of BP, zigzag and armchair, leading to anisotropy.^[^ ^44^, ^45^, ^46^, ^47^ ^]^ This is the most interesting structural feature of BP. It has been visualized using AC‐STEM on three different zone axes systematically.^[^ ^48^ ^]^ Owing to this structural anisotropy, it is demonstrated that intercalation of metallic atoms into BP was also anisotropic.^[^ ^49^ ^]^ Lee et al. showed Cu intercalation in BP using in situ STEM combined with DFT study. Such an anisotropy of intercalation induces semiconductor to semimetal transition of angstrom‐wide electronic channels in BP.^[^ ^49^ ^]^ Furthermore, Haratipour et al. investigated the relationship between atomic structure configuration and electronic mobility of BP as gate dielectrics.^[^ ^44^ ^]^ Srivastava et al. also investigated the relationship between atomic configuration and electronic structure of BP. In this study, BP in zigzag direction is sandwiched by BPs in armchair direction to construct resonant tunneling diodes.^[^ ^50^ ^]^ The degree of interlayer interaction strength is reliant on the twist angle between the BP layers. As a result, by adjusting the twist angle, it is possible to regulate the vertical transfer of electrons, which can range from ohmic to tunneling.^[^ ^51^ ^]^

TEM and AC‐STEM imaging and analysis of BPs were used to prove the existence of and show the morphology of BPs. We expect that the special structural anisotropy of BP can provide unique atomic configuration of defects and structure and property relationships in the future studies.

### 2.5 MXenes

**Figure 3:**
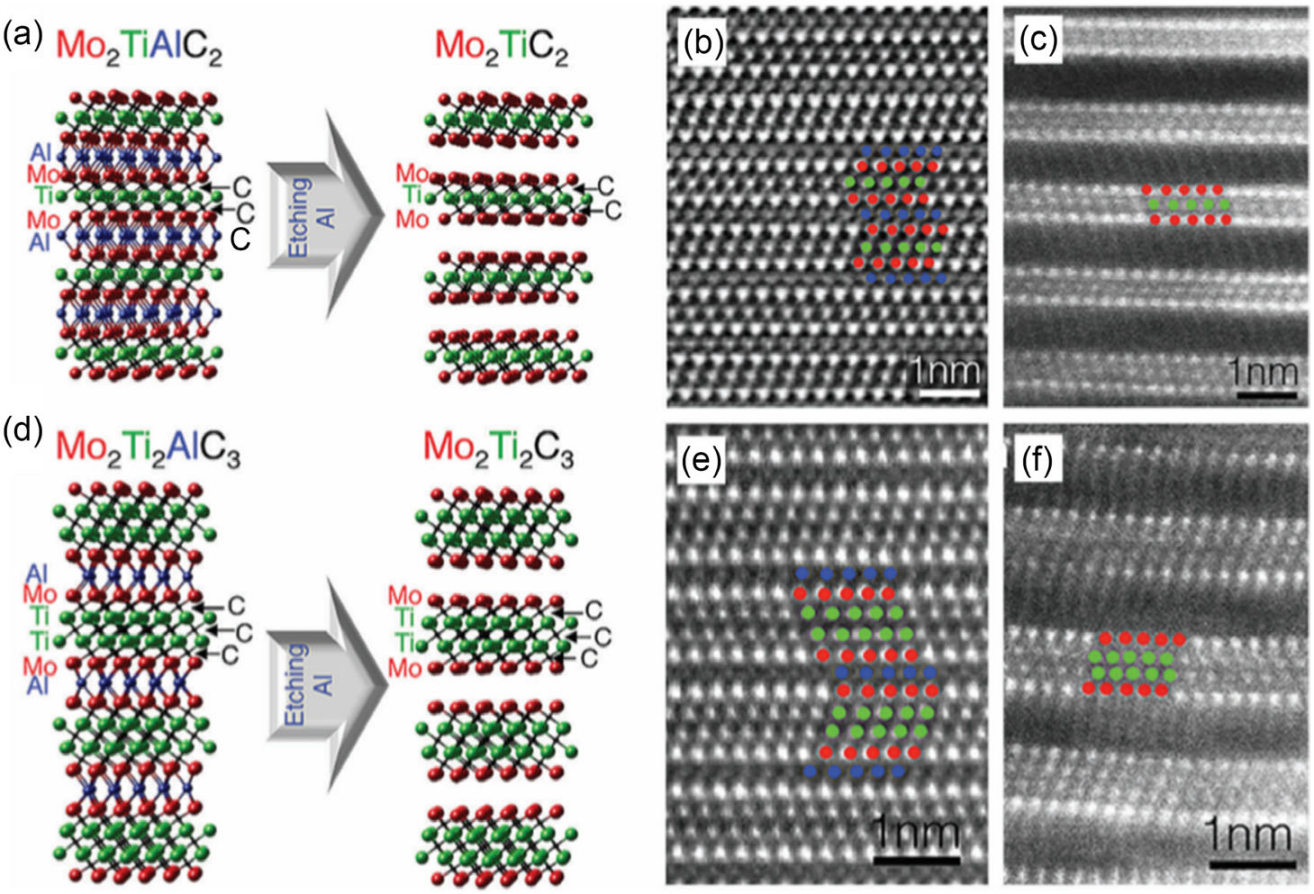
a) Images showing the atomic models of Mo_2_TiAlC_2_ and Mo_2_TiC_2_, along with cross‐sectional STEM images. The models use a color scheme to indicate different elements. b,c) STEM images corresponding to the atomic models of Mo_2_TiAlC_2_ and Mo_2_TiC_2_, respectively. The images are taken from a cross‐sectional view. d) Atomic models depicting Mo_2_Ti_2_AlC_3_ and Mo_2_Ti_2_C_3_, with the same color scheme to indicate different elements. e,f) The corresponding STEM images show cross‐sectional views of Mo_2_Ti_2_AlC_3_ and Mo_2_Ti_2_C_3_, respectively. a–f) Adapted with permission.^[^ ^54^ ^]^ Copyright 2015, American Chemical Society (https://doi.org/10.1021/acsnano.5b03591; further permissions for the material excerpted should be directed to the ACS).

MXenes are 2D layered materials composed of transition metal M, group 13/14 element A, and carbon or nitrogen X. The general composition of MXenes is M_*n*+1_AX_*n*_, where *n* can be 1, 2, or 3^[^ ^52^, ^53^ ^]^ MXene is schematically illustrated in **Figure** **3**,^[^ ^54^ ^]^ where the parent structure is transformed from M_*n*+1_AX_*n*_ to M_*n*_M_*m*_AX_(*n*+*m*)−1_ due to the inclusion of an additional metal. The removal of aluminum through selective etching can alter the layer structure of the material being imaged, resulting in the formation of MXenes by removing the A group from the parent MAX. The layered structure of MXenes lends them toward applications in energy storage, composites, catalysts, and electronic devices.^[^ ^55^ ^]^

AC‐STEM has been used to directly visualize the atomic‐scale defects in titanium carbide MXene (Ti_3_C_2_T_*x*_).^[^ ^56^, ^57^, ^58^ ^]^ Defect formation can be occurred in any of the three metal sublayers of the MXene monolayer, and the image contrast difference in AC‐STEM between the two vacancies indicates that they are in different layers. Eklund et al. argued that when the evolution of titanium vacancies occurs in the same sublayer, they form into a honeycomb configuration.

The DFT calculation results reveal that coalescing these vacancies in this manner has a minimal energy cost to the system. However, accurately determining formation energies for larger vacancy clusters is difficult due to limitations imposed by the input repeat‐unit supercell size and subsequent boundary interactions. According to a study by Karlsson et al., treatment with HF solution can lead to the formation of vacancies in Ti atoms, and the concentration of these vacancies may depend on the concentration of HF used in the treatment.^[^ ^57^ ^]^ Adatoms of Ti have a preference to be located on the basal planes of MXene layers. AC‐STEM imaging demonstrated that Ti adatoms in Mxene have the place above Ti atoms in the MXene layer.^[^ ^56^ ^]^ Electron energy loss spectroscopy (EELS)‐based elemental analysis revealed that oxygen‐containing surface groups are unevenly distributed on the basal surface of the MXene layer, which affects the valence of the local Ti atoms. Tao et al. showed the possibility of defect engineering to create a MXene with homogeneous defect distribution,^[^ ^59^ ^]^ where a regular superlattice of divacancies was introduced by designing an appropriate precursor MAX.

## 3 Atomistic Visualization of Defects and Dopants

In this section, we will discuss the atomic arrangements of 2D materials with defects and 2D material heterostructures. The categorization of defects includes three types: point defects, 1D defects, and 2D defects. When STEM imaging in the microscope is carried out, formation of the defects is significantly affected due to the electron beam radiation. This section will cover vacancies and impurities and their effect on the electromagnetic and optical properties. Electron beam irradiation gives a driving force to generate point defects. 1D defects include the line of vacancies and the GB. A line of vacancies can be created through the migration and aggregation of isolated vacancies. The configuration of GBs, on the other hand, is determined by the angle of the grain. These defects have significant impacts on the band structure of the 2D TMDs locally, and some 1D defects can have a role as metallic wires embedded in the semiconductor sheet matrix. In the next step, nanowires form, and holes open with the increased concentration of S vacancy lines increase. The 2D TMDs undergo brittleness and eventually break, leaving behind atomically sharp crack tips and long straight edges in the undamaged area.

### 3.1 Point Defects

Point defects are a class of 0D structural defect. In general, there are two types of point defects in 2D materials, which are vacancies and antisite defects. The formation of defect is very common in 2D materials.

Zhou et al. identified single defect in graphene and carried out low‐loss EELS analysis to measure plasmon resonance at the defect site, which shows localized plasmons.^[^ ^60^ ^]^ Robertson et al. carried out AC‐STEM imaging of defective graphene with mono‐, di‐, and multivacancies and suggested atomistic models of each case.^[^ ^61^ ^]^

**Figure 4:**
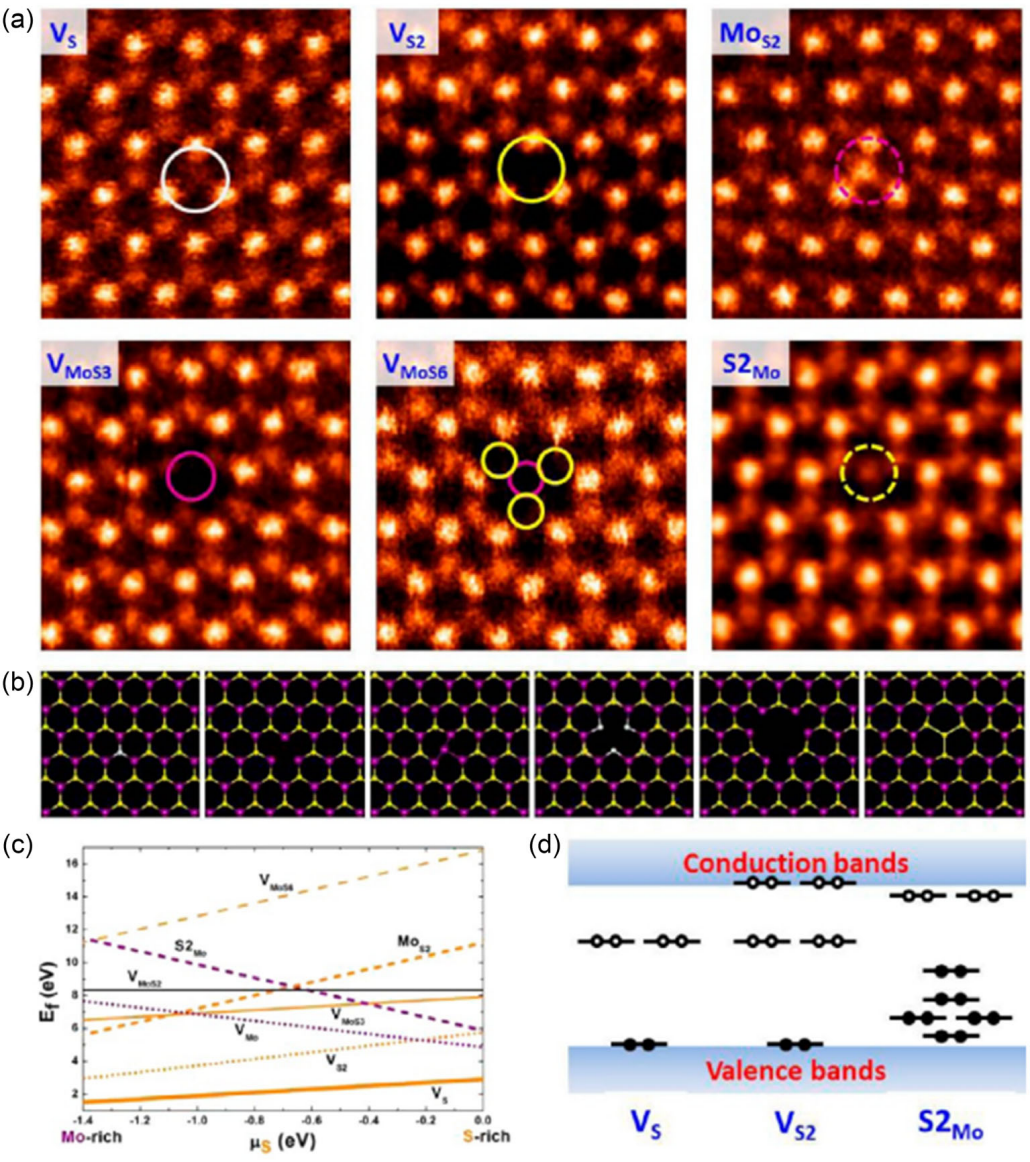
A) Atomic‐resolution ADF images of different types of intrinsic point defects in monolayer CVD MoS_2_, which include V_S_, V_S2_, Mo_S2_, V_MoS3_, V_MoS6_, and S2_Mo_. B) Fully relaxed structural models of the six types of point defects observed experimentally. From left to right: V_S_, V_S2_, Mo_S2_, V_MoS3_, V_MoS6_, and S2_Mo_. Purple, yellow, and white balls represent Mo, top layer S, and bottom layer S, respectively. C) Formation energies of different point defects as functions of sulfur chemical potential, plotted in the range −1.4 eV < *μ*_S_ < 0 eV, where MoS_2_ can remain stable with respect to the formation of bulk Mo (*μ*_S_ = −1.4 eV) or bulk alpha‐S (*μ*_S_ = 0 eV). D) Schematic representation of the defect levels. A–D) Adapted with permission.^[^ ^63^ ^]^ Copyright 2013, American Chemical Society.

In the case of monolayer MoS_2_, S atomic loss is most common and can occur from both top and bottom side of layers. This phenomenon is shown in **Figure** **4**A.^[^ ^62^, ^63^, ^64^ ^]^ Little strain or bond reconstruction occurs when a vacancy in a sulfur atom or molecule is formed.

Zhou et al. identified six distinct types of point defects in MoS_2_, which are frequently observed during the chemical vapor deposition (CVD) synthesis of MoS_2_, as illustrated in Figure 4A.^[^ ^63^ ^]^ Through quantitative analysis of the ADF STEM image intensity, AC‐ADF STEM imaging's atom‐by‐atom chemical analysis capability allows us to distinguish between a monosulfur vacancy and disulfur vacancy, as well as identify antisite defects from regular lattice sites with precision. Wang et al. used DFT calculations to determine the energy level of the observed point defects. The favorable structures of defects with low energy levels obtained from DFT calculations are shown in Figure 2b, and they are in excellent agreement with the experimental STEM images. Threefold symmetry is preserved in most of the defect configurations. In contrast, MoS_2_ breaks the symmetry explicitly by positioning Mo closer to two out of the three nearest‐neighbor Mo atoms and at one of the two S layers. According to the DFT calculation, the structural stability of diﬀerent point defects can be evaluated by calculating their formation energies. Figure 4C displays how defect formation energies vary as a function of the chemical potential of sulfur across a wide range of compositional conditions, ranging from Mo‐ to S‐rich and Figure 4D shows the energy level of defects. The gradient of the energy profiles is directly related to the changes in the concentration of sulfur atoms within the defects. Moreover, the formation energy of disulfur vacancies is roughly twice that of monovacancies of sulfur, indicating that it is hard for sulfur monovacancies to combine. The experimental observations also confirm the theoretical result, as randomly distributed monovacancies in sulfur were observed more frequently than divacancies in sulfur. Acerce et al. reported that in the case of graphene, the opposite phenomena are observed. That is, divacancies are energetically preferable to monovacancies.^[^ ^64^, ^65^ ^]^

### 3.2 Dopants

In this section, foreign atoms at the interstitial site or doping in chalcogenide or metal sites in TMDs will be covered. The usage of AC‐STEM and EELS to identify dopant elements and the high‐temperature stability of dopants will be also investigated. Extrinsic defects in TMDs can be classified into two types based on the location of foreign atoms relative to the 2D lattice. One is “dopants in lattice” and the other is denoted as “dopants on the surface.” Regarding “dopants on the surface”, dopant atoms either reside on the surface of pristine 2D TMD crystals or become trapped within structural imperfections like vacancies. They can also act as adatoms due to the heightened reactivity in areas with unsaturated bonds or strain fields caused by defects.

In general, there are two required conditions for the successful and stable incorporation of foreign atoms into the TMD lattice. First, the atomic radius of the dopant should be close to that of the parent atom. Second, even if the doping level gets high, the lattice framework should not be changed. Thus, substitutional foreign atoms positioned near the transition metal or chalcogen elements of 2D TMDs in the periodic table are the most suitable options. Both the transition metal and the chalcogen atoms are substituted by other elements in the same group of the periodic table with similar radius, valence, and coordination manner. Monolayer Mo_1−*x*_W_*x*_S_2_,^[^ ^66^, ^67^, ^68^, ^69^ ^]^ MoS_2(1−*x*)_Se_2*x*_,^[^ ^70^, ^71^ ^]^ and WS_2(1−*x*)_Se_2*x*_^[^ ^72^ ^]^ with *x* varying from 0 to 1 are the typical examples of doped 2D TMDs. They are prepared by atomic layer deposition (ALD), CVD, and other growing techniques. When examining doped 2D TMDs, the primary goals are 1) determining the substitutional site within the TMD lattice; 2) identifying the crystal phase of TMD alloys; and 3) assessing how dopants are distributed within the alloy lattice.

In case of MoS_2(1−*x*)_Se_2*x*_ (Se‐doped MoS_2_) monolayers, both S and Se atoms occupy the chalcogen sites while Mo atom sites are not shared and^[^ ^70^, ^71^ ^]^ thus, MoS_2(1−*x*)_Se_2*x*_ has only the 1H phase. Four types of atomic arrangements exist in MoS_2(1−*x*)_Se_2*x*_; Mo, S_2_, SSe and Se_2_, respectively. Such arrangements indicate the uneven distribution of the atomic column intensity at four diﬀerent regions in the intensity histogram.^[^ ^71^ ^]^ The arrangement of three chalcogen pairs around Mo atoms, with varying numbers of Se dopants, was observed to conform to the binomial distribution expected from the Se content. This suggests that the configurations are randomly arranged based on the probability of certain configurations under the assumption of a random distribution of S and Se atoms. It indicates the evenly mixture between S and Se in MoS_2(1−*x*)_Se_2*x*_ monolayers.^[^ ^70^ ^]^ Gong et al. conducted a similar analysis on Se‐doped MoS_2_ with an AB stacking sequence and reported an odd distribution of S and Se atoms.^[^ ^71^ ^]^

Lin et al. demonstrated that molybdenum and tungsten ditelluride alloyed with S or Se atomic layers (MX_2*x*_Te_2(1−*x*)_, M = Mo, W, and *X* = S, Se) result in phase‐dependent ordering. In case of 1T′‐phase material, MX_2*x*_Te_2(1−*x*)_ shows anisotropic ordering of chalcogen atoms, while the distribution of alloy atoms in the 2H‐phase is uniform in all directions.^[^ ^72^ ^]^

Doping of transition metal with different electron configurations into 2D TMDS shows interesting behaviors. Manganese (Mn) and rhenium (Re) atoms have one additional valence electron compared to Mo or W atoms because they are in group 7 in periodic table. Incorporating atoms such as Mn and Re, which have one extra valence electron compared to Mo or W atoms, into monolayer MoS_2_ can potentially result in n‐type doping of the material. Zhang et al. reported that Mn atoms, exhibiting 50% atomic column intensity compared with Mo in the probe‐corrected HAADF‐STEM image, not only disperse in the pristine MoS_2_ lattice, but also segregate into GBs, acting as substitutional dopant atoms in the place of Mo. Zhang et al. also demonstrated that whether Mn doping of MoS_2_ is successful or not significantly depends on the substrates. Doping of MoS_2_ with Mn has been successful on inert substrates, such as graphene, while it is not possible to dope the material on the substrates with reactive surface terminations (e.g., SiO_2_ and sapphire).^[^ ^73^ ^]^ Lin showed that in case of Re‐doped MoS_2_, most Re dopant atoms at the Mo sites (Re@Mo) are stable under the electron beam irradiation for a long period (240 s).^[^ ^74^ ^]^ A minority of Re atoms, which are mobile under the electron beam illumination, move on the surface of MoS_2_ lattice as adatoms. These Re atoms are prone to residing on S sites beside the Re@Mo sites to form a complex of Re–S + Re@Mo.^[^ ^74^ ^]^ Gao et al. reported that incorporation of substitutional Re atoms does not change the structure of MoS_2_ and just leads to n‐type doping.^[^ ^75^ ^]^ Nb atoms are doped in the WS_2_ lattice with various atomic arrangements including nonbonded single Nb atoms, small Nb clusters, and 1D chain of Nb atoms, maintaining the structure of WS_2._ On the other hand, the crystal structures formed by Mn bonding with S, such as rock‐salt, zinc‐blende, or wurtzite, and ReS_2_'s distorted octahedral structure are all non‐2D and have significantly different structures compared to the host TMD material.^[^ ^68^, ^69^ ^]^ In a separate study, Nb dopants were found to cause a redshift in the PL peak without compromising the direct bandgap feature of monolayer WS_2_, as reported by Sasaki et al.^[^ ^76^ ^]^

All dopants mentioned above were substituted intentionally to provide the various electronic properties of TMD materials. However, the introduction of unexpected impurities can compromise the preservation of the pristine states and properties of TMDs. In a study conducted by Robertson et al., it was discovered that individual chromium (Cr) and vanadium (V) atoms can effectively act as stable substitutional dopants. These dopants can replace molybdenum (Mo) atoms within monolayer MoS_2_ that is synthesized using CVD process.^[^ ^77^ ^]^ Their elements were identified by ADF‐STEM with combination of the atomic‐resolution EELS.^[^ ^77^ ^]^

Dopant atoms with atomic sizes or coordination numbers that do not closely match those of the host 2D TMD crystal, such as Pt and Au, are more likely to be localized on the surface of TMD materials rather than being incorporated into lattice sites. This localization can result in lattice strain and out‐of‐plane distortion. Li et al. revealed that in the case of monolayer MoS_2_, isolated Pt atoms prefer to locate on S vacancy sites on the clean surface.^[^ ^78^ ^]^ The low migration energy barriers across the MoS_2_ surface enable isolated Pt atoms to be highly mobile, making them capable of easily hopping between different S vacancy sites under electron beam irradiation. In contrast, when there is amorphous hydrocarbon contamination on the MoS_2_ surface during AC‐STEM observation, the single Pt atom cannot bind to the surface because its configuration is determined by the bonding between Pt and carbon rather than the interaction between Pt and the MoS_2_ lattice. Liu et al. studied single Co dopants in monolayer MoS_2_ for the design of new hybrid catalyst systems. The most energetically favorable place of single Co dopants is MoS_2_ basal plane and edges. The study by Liu and co‐workers utilized AC ADF‐STEM and EELS to demonstrate that Co atom dopants can attach to various surface sites, such as those located atop Mo and in the hollow center of hexagonal rings. This highlights the energetic favorability of accommodating Co dopants in numerous unsaturated basal S vacancies in MoS_2_.^[^ ^79^ ^]^ In contrast, the stability of Au atoms on the MoS_2_ surface is low, and they tend to be located on top of Mo, S, and the hollow center of MoS_2_ hexagons. It has been reported that only a small fraction of Au atoms acts as single substitutional dopants, as Au occupying the Mo site is not energetically favorable. Instead of uniformly distributed, Au adatoms have tendency to aggregate. The diffusion rate of Au adatoms on the MoS_2_ surface is high, which makes it difficult to fully distinguish the kinetic pathways involved.^[^ ^74^ ^]^ The arrangement of Au adatoms is comparable to that of Mo single atoms on the MoS_2_ monolayer in its native state. Hong and co‐workers employed time‐resolved ADF‐STEM imaging with an interframe time of 3 s to analyze the migration path of the individual Mo atom. They observed that the Mo atom migrated step by step across sites on top of Mo and the hollow center of hexagons without any direction preference. This research may serve as a reference for exploring the dynamics of Au adatoms on TMDs.^[^ ^80^ ^]^ When the amount of adatoms increases, the adatoms become aggregated clusters on the TMD surface, whose arrangement are determined by the interaction between the cluster and the TMD lattice underneath as well as the structural nature of the bulk materials. In a recent study utilizing ADF‐STEM, the formation of Pt nanocrystals was monitored using an in situ heating holder while depositing Pt adatoms at a temperature of 800 °C.^[^ ^81^ ^]^ Despite the bulk Pt adopting a 3D face‐centered cubic crystal structure, the deposition of Pt adatoms on monolayer MoS_2_ templates enabled stabilization of Pt nanoclusters consisting of approximately 20 atoms into a single atomic plane. This indicates that arrangement of clusters is strongly affected by the TMD lattice. Beyond a certain critical number of Pt atoms, the particles adopt a 3D face‐centered cubic structure, eliminating the effect of the MoS_2_ template on the lattice constant of Pt nanoparticles. In a study by Wang et al., the interaction between the surface of monolayer MoS_2_ and CuCl nanoclusters resulted in the formation of monolayer crystalline CuCl nanoclusters, which are ionic crystals with a 3D cubic zinc blende structure in bulk.^[^ ^82^ ^]^ Similar to Pt clusters, they exhibited preferential epitaxial alignment with MoS_2_ in two distinct orientations and underwent discrete rotation on the surface when subjected to electron beam radiation. In addition, a DFT investigation revealed a strong chemical bond between the 2D CuCl nanocrystals and monolayer MoS_2_. This interaction significantly impacts the material's overall properties, resulting in a type‐III band alignment and metallic behavior for this hybrid system, regardless of the stacking pattern. The investigations offer valuable insights into the influence of dopant atoms on the structure of 2D layered materials and the interaction between dopants and 2D materials. Identifying the exact position of dopants on 2D materials has provided the structural base for calculating electronic structures. Therefore, it became possible to analyze changes in electron density, changes in electronic structure, and consequent electromagnetic properties by doping elements.

### 3.3 1D and 2D Defects

1D defects are defined as a series of linearly extended defects. Their configurations are far from the long‐range periodicity in the pristine 2D material lattice. For monolayer 2D TMD materials, defects with 1D structure can be categorized into two types depending on their effect on the lattice orientation. The first type, referred to as common line defects (c‐LDs), has the same crystal direction on both sides of the defect. The second type, known as GBs, has a difference in crystal orientations on both sides of the defect.

**Figure 5:**
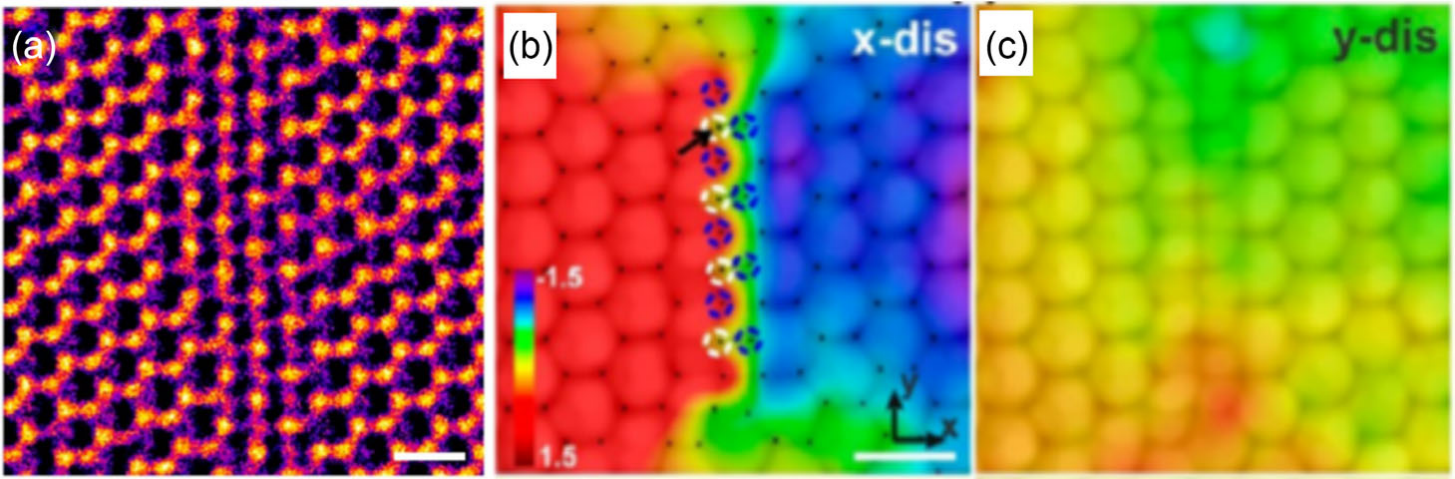
a) AC‐TEM image of line vacancies in a monolayer of MoS_2_ that resulted from electron beam irradiation at 80 kV. b,c) Displacement maps across the 1S line vacancy in both the *x* and *y* directions. Scale bar: 0.5 nm. a–c) Adapted with permission.^[^ ^65^ ^]^ Copyright 2016, American Chemical Society.

Generally, monosulfur vacancies are mobile and easily diﬀuse within MoS_2_ due to the low energy barrier for migration under electron beam irradiation. They tend to aggregate into c‐LDs, as shown in **Figure** **5**a. When the length of c‐LD increases, missing of S atoms induces the lattice compression within the central region of the c‐LD. An atomic displacement mapping was performed on a 1S line vacancy in a MoS_2_ monolayer along both the perpendicular (*x*) and parallel (*y*) directions of the vacancy, revealing lattice compression in the × direction (see Figure 5b,c). The S vacancy lines stagger top and bottom to stabilize the system and reduce buckling. The width of linear vacancies has a capability to tailor the band structure and electronic properties, which shift from semiconductor to metallic. The above sentence suggests that the presence of linear vacancy defects in the semiconducting monolayer MoS_2_ can create metallic channels.^[^ ^83^ ^]^ This is because the S linear vacancy defects cause compression strain locally around their position. However, as there are multiple c‐LDs along different zigzag angles, there are also competing forces at play. Lin et al. argued that owing to the trigonal symmetry of monolayer 1H MoS_2_, the strain from one c‐LD is not completely orthogonal to the other c‐LD. Due to local sulfur deficiency and the formation of triangular domains at the intersection of three linear vacancies, inversion domains are formed.^[^ ^84^ ^]^ This type of line defect is typically observed at high temperatures where sulfur vacancy diffusion is thermally activated.

A GB is another type of 2D defect in 2D materials. GB formation occurs through the atomic stitching between two different grains. Huang et al. clearly identified the exact configuration of C atoms at the GBs of graphene in combination of grain size mapping, which demonstrated that GBs strongly affect mechanical strength, but not electrical properties.^[^ ^11^ ^]^ Namejei et al. found that GBs in 2D TMD materials can take the form of dislocation cores, which are separated by a distance that depends on the relative orientations of the adjacent grains.^[^ ^85^, ^86^ ^]^ These dislocation cores are composed of fivefold and sevenfold (5|7) rings, 4|6 rings, 6|8 rings. Those rings include substitution between metal, chalcogen, or carbon atoms.^[^ ^17^, ^85^, ^86^ ^]^ Furthermore, the dislocation cores observed in MoS_2_ exhibit periodic gaps, which are also evident in the GB areas.^[^ ^61^ ^]^

**Figure 6:**
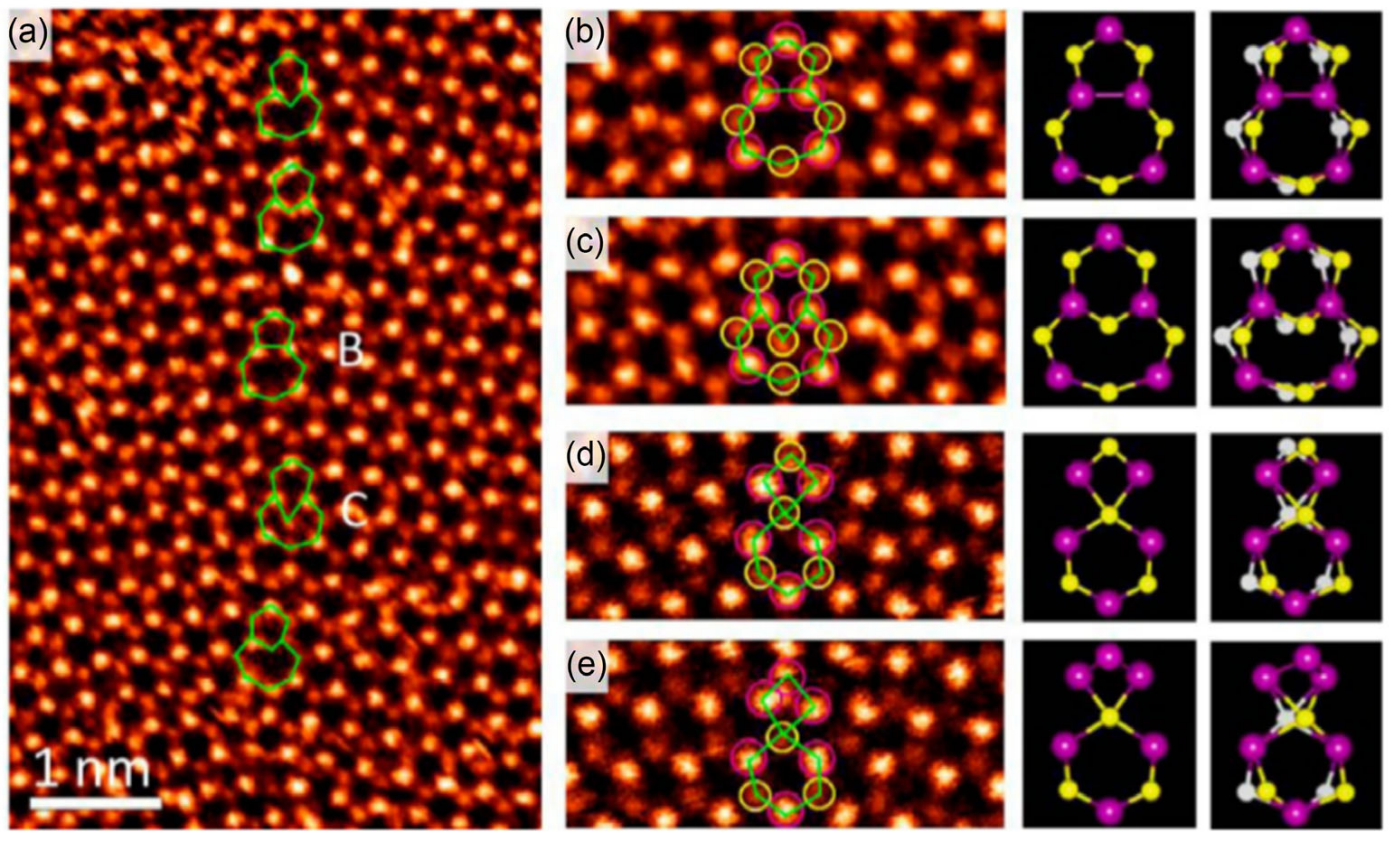
The atomic structure of small‐angle GBs in monolayer MoS_2_. a–c) STEM‐ADF images of an 18.5° GB made up of dislocations with fivefold and sevenfold rings (5|7) and dislocations with sixfold and eightfold rings (6|8). b,c) A zoomed‐in view of the 5|7 and 6|8 structures from the regions highlighted in (a). d,e) ADF images of a 17.5° GB consisting of dislocations with fourfold and sixfold rings (4|6), either pristine (d) or with Mo‐substitution (e). The 2D and 3D structural models for the various dislocation structures are placed next to the corresponding ADF images. a–e) Adapted with permission.^[^ ^63^ ^]^ Copyright 2013, American Chemical Society.

**Figure** **6**a displays a GB that is titled at an angle of 18.5°, featuring dislocation cores of 5|7 and 6|8 types. Further atomic details of these dislocation cores are presented in Figure 6b,c, respectively. The 5|7 core serves as the fundamental dislocation core structure, while the addition of S or 2S into the Mo—Mo bonds results in the emergence of 6|8 structures observed in the same GB. On the other hand, different 4|6 dislocation cores along the same GB were observed (see Figure 6d,e) with fourfold and sixfold rings joined by fourfold coordinated S atoms. The 4|6 structure, as shown in Figure 4D, can be derived from 5|7 structures by removing 2S atoms. In case of 60  GB in MoS_2_, antiphase boundary has been formed and it induces a mirror symmetric domain.^[^ ^87^ ^]^ van der Zande et al. reported that the edge‐sharing 4‐ and 8‐member rings can join the antiphase domains, which resembles previous study.^[^ ^88^ ^]^ Regarding the bilayer system, the overlaid atomic structures disclose two distinctive phases due to different stacking sequences (2H and 3R), particularly when one layer is superimposed on top of the other layer including the antiphase boundary.

## 4 2D Heterostructures

The creation of 2D heterostructures has become increasingly popular because of their distinct characteristics that are not present in single compound of 2D layered materials. Additionally, these heterostructures have the potential to be used in electronic and optoelectronic devices.^[^ ^89^, ^90^ ^]^ In spite of the van der Waals interaction being the dominant force between the layers of 2D materials, it is important to note the presence of interlayer interactions and couplings that are observed in 2D heterostructures, which encompass van der Waals forces as well.

**Figure 7:**
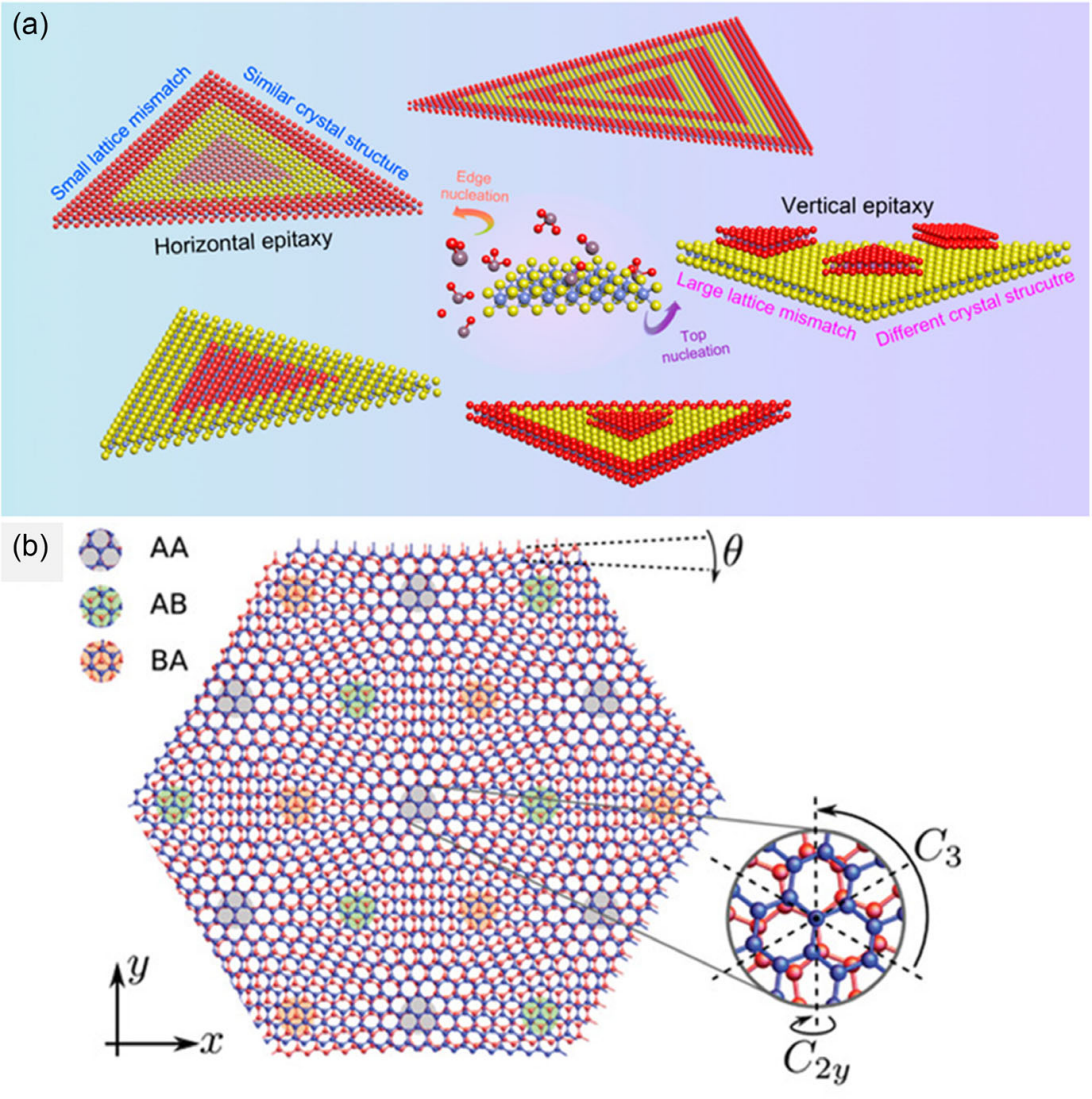
a) Schematic illustration of horizontal and vertical heterostructures. Adapted with permission.^[^ ^91^ ^]^ Copyright 2022, American Chemical Society. b) Moire patterns of 2D materials. Adapted with permission.^[^ ^92^ ^]^ Copyright 2018, American Physics Society.

Interestingly, unlike STEM observation of other materials, atomic‐resolution visualization of 2D heterostructures includes in‐plane and vertical types on plan‐view and cross‐sectional view, which gave us meaningful information. In this section, atomic observation of two types of 2D heterostructures—vertical and horizontal heterostructures—and Moire patterns is going to be introduced. A schematic description of each heterostructures is illustrated in **Figure** **7**.^[^ ^91^, ^92^ ^]^

### 4.1 Vertical Heterostructures

The first successful synthesis of vertical heterostructure was reported by Ajayan et al. WS_2_/MoS_2_ and WSe_2_/MoS_2_ vertical junctions have been built up by one‐ and two‐step CVD routes and show intriguing electromagnetic and optical properties.^[^ ^93^ ^]^ ADF‐STEM study showed 2H stacking behavior of the WS_2_/MoS_2_ heterostructure. In 2017, Pan et al. reported a two‐step growth of vertical WSe_2_/SnS_2_ heterostructures. Although the authors carried out structural analysis using high‐resolution TEM, Moire lattices are observed on plan view.^[^ ^94^ ^]^ Similar Moire superlattice which is formed due to lattice mismatch has been observed in the GaSe/MoSe_2_ heterostructure.^[^ ^95^ ^]^ The GaSe/MoSe_2_ heterostructures generate p–n junctions that effectively convey and segregate photogenerated charge carriers, which leads to a controllable photovoltaic response through a gate. Defect structure of atomic interfaces has been visualized using ADF‐STEM by comparing calculated and experimental interlayer distance.^[^ ^96^ ^]^ While the thickness of h‐BN‐encapsulated WSe_2_ is decreased from bulk to monolayer, interlayer separation was systematically increased, which is attributed to flexible and easily deformed transitional metal chalcogenide monolayer.

### 4.2 Horizontal (In‐Plane) Heterostructures

**Figure 8:**
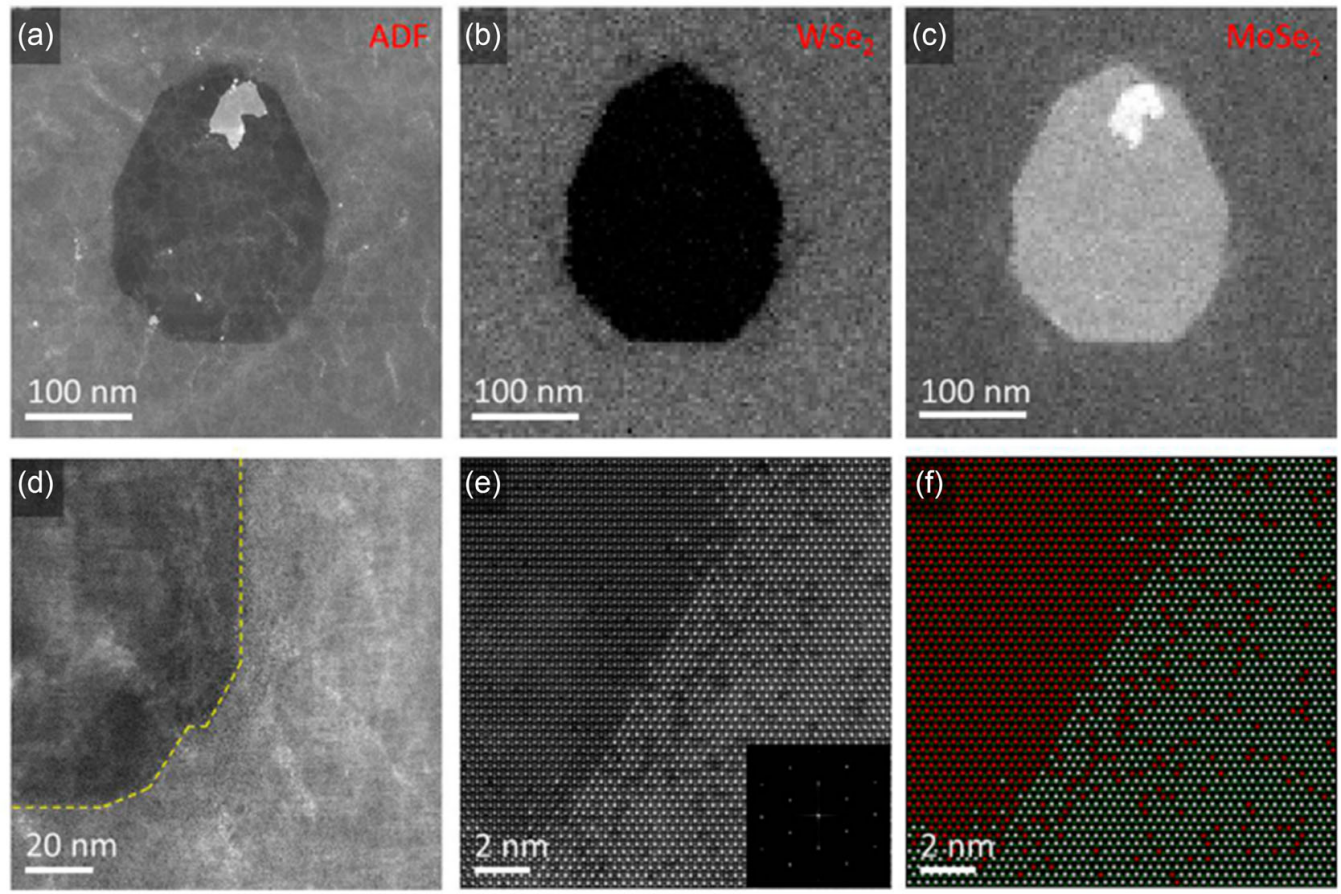
STEM imaging and elemental mapping of lateral WSe_2_/MoSe_2_ heterostructures. A) Low‐magnification STEM annular dark‐field (ADF) image displaying a lateral WSe_2_/MoSe_2_ heterostructure grown on a large MoSe_2_ monolayer. B,C) Corresponding maps of WSe_2_ and MoSe_2_ in the region highlighted in (A), illustrating that the second MoSe_2_ layer acts as the nucleation center for epitaxial growth of WSe_2_. WSe_2_ is observed only in the brighter outer region depicted in the ADF image, while the MoSe_2_ map reveals the presence of a small second layer atop a large monolayer. D,E) Higher‐magnification STEM ADF images of the lateral interface depicted in (A). Inset in (D): FFT of the ADF image demonstrating lateral epitaxy between WSe_2_ and MoSe_2_. F) Structural model of the top layer's lateral interface, derived from atom‐by‐atom quantitative image analysis of the ADF image shown in (E). The white spheres represent W atoms, red spheres depict Mo atoms, and dark green spheres denote Se atoms. a–f) Adapted with permission.^[^ ^98^ ^]^ Copyright 2015, American Chemical Society.

Stitching together edges of different types of 2D materials forms lateral heterostructures, which facilitate precise tuning of the band offset due to the greater spatial separation of materials within the heterostructure. However, building up lateral heterostructures still remains tough and challenging because TMD alloys are thermodynamically stable than lateral heterostructure. Recently, synthesis of MoS_2_–MoSe_2_,^[^ ^97^ ^]^ WS_2_–MoS_2_,^[^ ^93^ ^]^ and WSe_2_–MoSe_2_^[^ ^98^ ^]^ lateral heterostructures were reported. **Figure** **8**A–f shows the epitaxial WSe_2_/MoSe_2_ heterostructures with elemental mapping and structural model.

The formation of a p–n junction within a monolayer thickness is effortless in lateral TMD heterostructures, offering the possibility of fabricating 2D ultrathin electronic devices. This is a main difference from vertical heterostructures. At the same time, a lateral heterostructure can maintain the excellent pristine properties of each material.

As mentioned above, studies on 2D heterostructures are focused on development of synthesis and measurement of physical properties, and Cs‐corrected STEM imaging has been usually used for confirmation of constitutional elements and crystal phases. STEM study combined with EELS analysis has become more important for revealing interfacial coupling, interaction, and thereby influences on physical properties of 2D heterointerfaces.

## 5 In Situ Observations

When the observation of 2D materials is carried out, 2D materials are exposed to electron beam inside transmission electron microscope with high energy.^[^ ^99^, ^100^ ^]^ Due to its low mass, an incident electron can be easily deflected by the atomic nucleus (elastic scattering) or by electrons surrounding the nucleus in the specimen (inelastic scattering) through Coulomb interactions. These interactions can have adverse effects such as ionization damage (radiolysis), displacement damage, and sputtering, which can result in atom loss. In this section, in situ observation of the phase transformation, defect formation, and electron beam‐driven crystallization of 2D materials using a scanning transmission electron microscope will be dealt with. In situ observation using conventional TEM is not considered.

### 5.1 Distortion‐Induced Transformation

Even if there is no atom loss while the observation is carried out, irradiation by electron beam can introduce distortions, resulting in local strain in the layered 2D materials. The atomic arrangements in the 2D material with a distorted structure change to relieve stress, resulting in a phase transformation. Lin et al. reported the observation of an in situ structural transformation between 2H and 1T phases in monolayer Re‐doped MoS_2_ under electron beam irradiation in the temperature range of 400–700 °C.^[^ ^101^ ^]^ The continuous bombardment of electrons induces thermal excitation and activates Re‐doped MoS_2_, enabling the displacement of atoms, which cause phase transition of the Re‐doped MoS_2_. This transformation led to the formation of the α‐phase, which was induced by the sliding of S layers and composed of three to four narrow zigzag chains that had a high affinity to nucleate at the sites where Re was substituted. The atoms at the corner of the Re‐doped MoS_2_ readily glide to release the local strain.^[^ ^101^ ^]^ STEM offers the advantage of being able to easily control the electron beam scanning area and irradiation time, making it possible to control the size and area of the phase transition. The continuous electron irradiation usually induces atomic loss, and therefore, formation of vacancies.^[^ ^102^, ^103^, ^104^ ^]^ The local strain caused by the atomic loss provides driving force stimulating the phase transformation. Elibol's study showed that when subjected to electron irradiation, 1H‐MoTe_2_ undergoes partial conversion into 1T′‐MoTe_2_, indicating a low phase transition barrier and the energetic preference of the 1T′ phase under strain.^[^ ^105^, ^106^ ^]^

**Figure 9:**
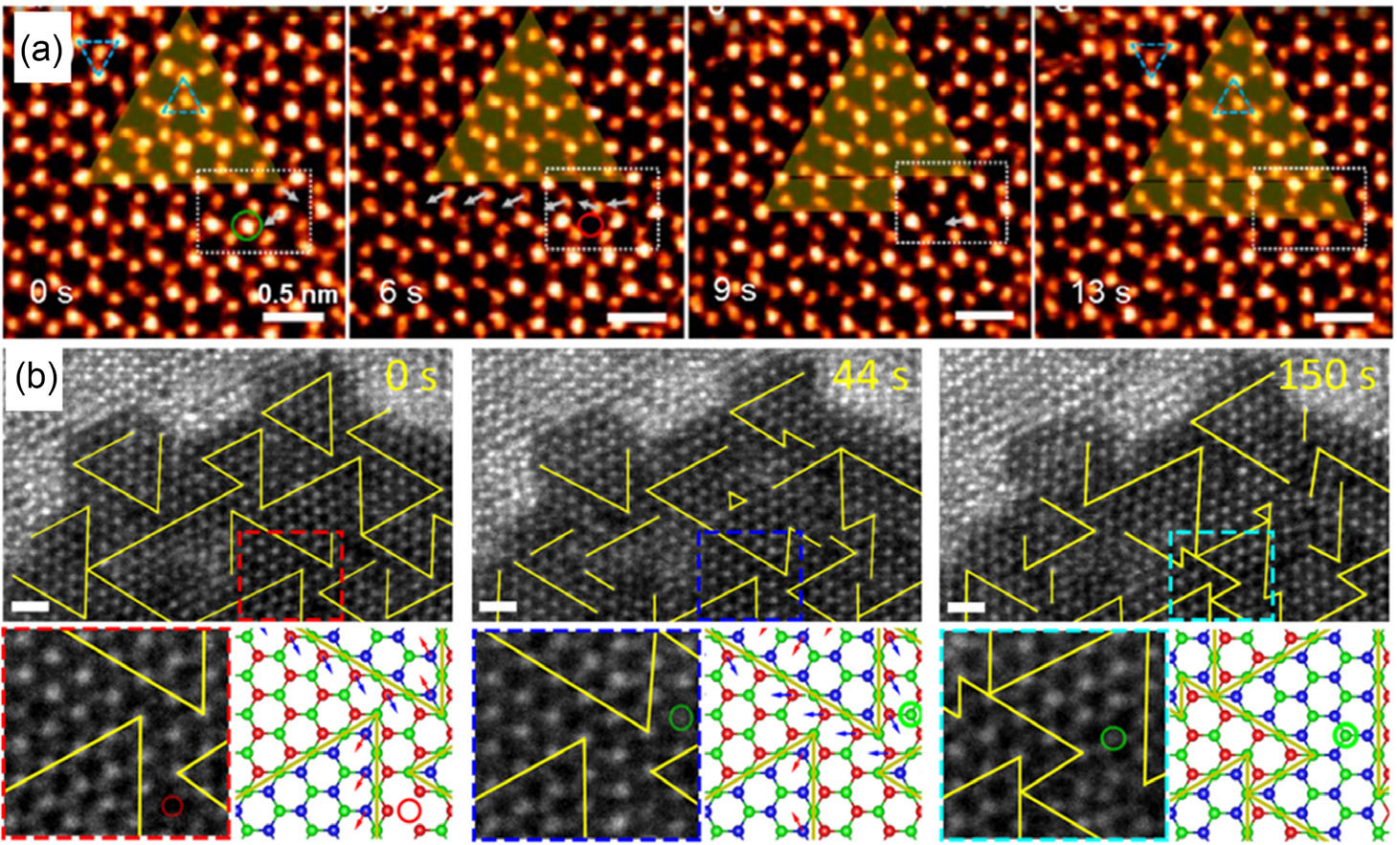
Distortion‐induced formation of new phase or domain. a) STEM image series showing the growth of the inversion domain. Adapted with permission.^[^ ^84^ ^]^ Copyright 2015, American Chemical Society. b) Temporal STEM image series showing the fast transformation of inversion domain boundaries upon reannealing at 250 °C with corresponding atomic models. Adapted with permission.^[^ ^106^ ^]^ Copyright 2017, American Chemical Society.

The vacancies easily migrate and agglomerate into extended defects under electron beam irradiation and excitation,^[^ ^60^, ^84^ ^]^ which is expected to play a critical role in the formation of nanodomains of 2D materials. To confirm the role of Se vacancies in the formation of GB and inversion domains, Zhu et al. used the electron beam to generate and excite Se vacancies within a monolayer MoSe_2_ and simultaneously monitored the dynamical structural evolution.^[^ ^84^, ^106^ ^]^ The movement of the 60° GB is triggered by the creation of vacancies in the Se atoms (highlighted by the circle, see **Figure** **9**a). Similar phenomena occurred during vacuum annealing when sufficient thermal energy is provided, as shown in Figure 9b.^[^ ^106^ ^]^ According to the studies, electron beam has been found to control nucleation, while thermal treatment is employed to promote growth. While these findings occurred within a microscope, these studies can provide insights into the growth mechanism of 2D materials due to the similarity between the microscopic environment and the synthetic conditions.

### 5.2 Vacancy Formation

Electron beam irradiation in a transmission electron microscope has the capability to produce and fabricate vacancies in 2D materials at various sites, which provides the driving force for defect control in TMDs and tailors the electronic properties of the 2D materials. The destabilization of Mo–S bonding caused by electron beam irradiation leads to the displacement of S atoms toward the surface, where they diffuse and create vacancy sites.^[^ ^107^, ^108^ ^]^ This kind of electron beam‐induced defect formation depends on electron dose. As time of irradiation becomes longer, electron beam sputtering causes nanopores to form in monolayer TMDs, while shorter irradiation causes the loss of just single metal atom. The ejected Mo atoms of the nanopore are easily absorbed on the MoS_2_ lattice close to the nanopore rather than being expelled totally outside, as the former structure is energetically favorable. By utilizing the AC‐STEM system and precise control of the angstrom sized electron probe, nanopores can be created with nearly uniform size and arranged into arrays with a 5 nm spacing through real‐time tracking of the electron‐drilling process. This suggests that this technique has the potential for generating an antidot lattice.

**Figure 10:**
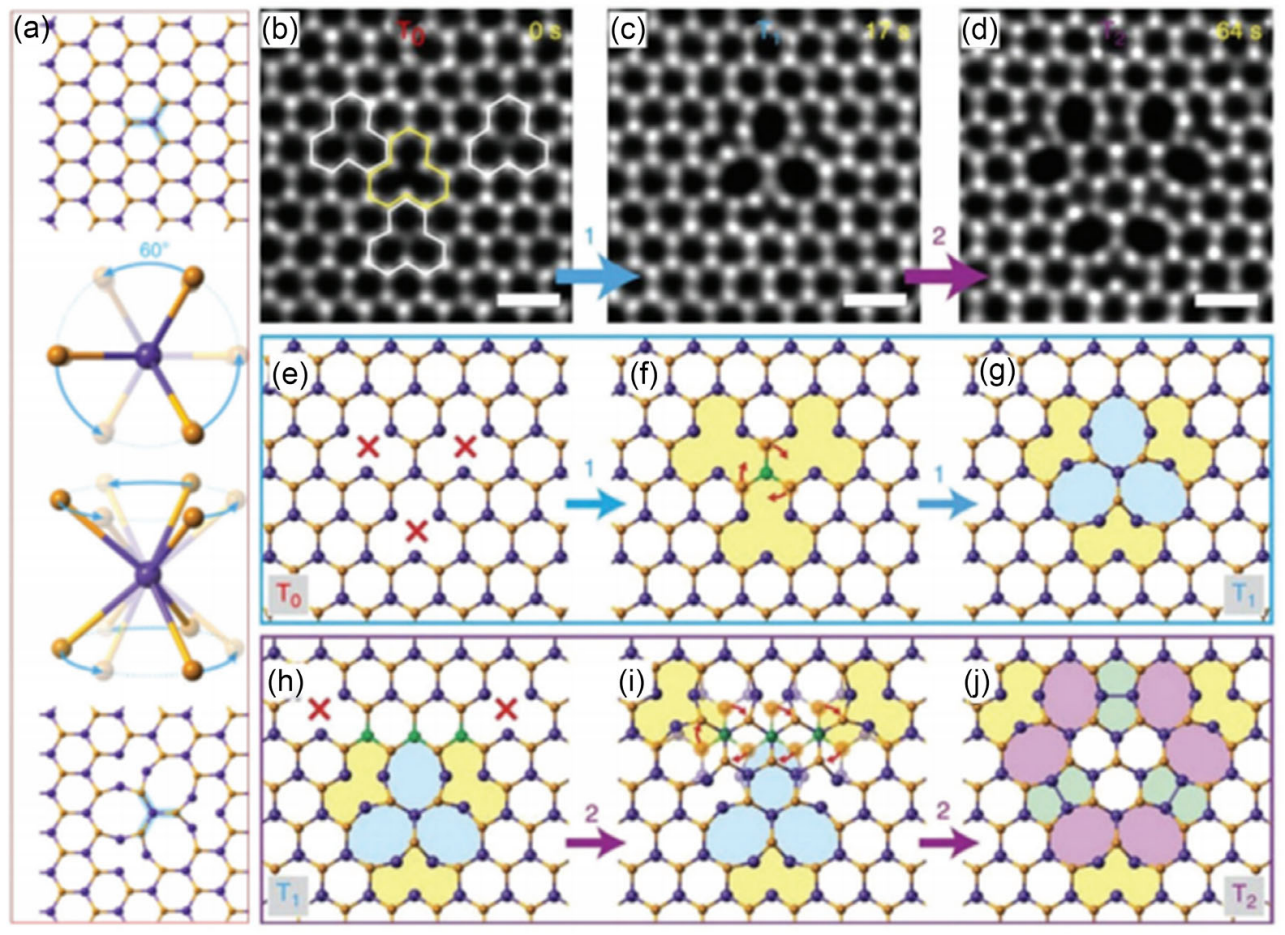
a) Schematic diagram to demonstrate the rotation of triple bonds between metal and chalcogen atoms by 60°, resulting in the formation of a refoil defect. b–d) A set of images obtained using ADF‐STEM show the formation of a trefoil defect in WSe_2_. Additionally, atomic models are presented in (e–j), which illustrate the bond rotation mechanism that leads to the formation of the trefoil defect. a–d) Adapted under the terms of the CC‐BY Creative Commons Attribution 4.0 International license (https://creativecommons.org/licenses/by/4.0).^[^ ^112^ ^]^ Copyright 2015, The Authors, published by Springer Nature.

Another notable aspect of in situ defect formation involves the generation of point defects through bond rotation in 2D TMDs. Defects can be formed in graphene through bond rotations taking place at any location.^[^ ^109^, ^110^, ^111^ ^]^ The presence of dopants has been observed to stabilize vacancies in graphene and has been found to be significant in the movement of vacancies. However, in 2D materials with binary compounds such as TMDs, bond rotation is much less common. Lin et al. observed rotation of bonding in monolayer WSe_2_ at elevated temperatures, which possibly cause p‐type doping and local magnetic moments.^[^ ^112^ ^]^ The defects in question are maintained in threefold symmetry, as depicted in **Figure** **10**a, through the rotation of three pairs of W–Se bonds centered on the same metal atom by 60°. Figure 10b–d shows the series of ADF‐STEM images showing the process of Se vacancies aggregation followed by transformation into the defect, resulting in rotation of bonding. Figure 10e–j describes the mechanism of bond rotation step by step. It indicates that bond rotations are present in 2D crystals with binary compounds, but it is not widespread. Moreover, the bond rotation is specific to WSe_2_.

### 5.3 Atomic Loss‐Induced Transformation

Ta et al. reported the stoichiometry‐dependent phase transformation gets easier if the inhomogeneous elemental loss ratio under electron irradiation makes the stoichiometry of the sample different.^[^ ^113^ ^]^ Layered tin dichalcogenides, for example, the controllabe loss of chalcogen atoms under electron irradiation, results in complete conversion to highly anisotropic orthorhombic tin monochalcogenides.^[^ ^114^ ^]^ Furthermore, Lin et al. presented another intriguing case of transformation induced by atomic loss, where bilayer PdSe_2_ was transformed into monolayer Pd_2_Se_3_ through interlayer melding and ejection of Se atoms under electron beam irradiation.^[^ ^115^ ^]^ Upon electron irradiation of layered PdSe_2_, Se atoms are evaporated, causing some Pd atoms to lose their bonds with Se. These Pd atoms then form a chemical bond with the nearest Se atom in the adjacent layer, leading to a decrease in the interlayer distance of PdSe_2_. As the concentration of Se vacancies increases, this eventually results in the melding of the two layers into one when the interlayer distance reaches a value close to the typical length of a Pd—Se bond.^[^ ^108^ ^]^

### 5.4 Cracks and Edges Formation

The continuous interaction of electron beam with MoS_2_ makes the line of S point defects, MoS nanowires, and holes. As a result, the formation of cracks takes place and these cracks originate from the hole. Wang et al. documented the propagation of these cracks along the zigzag direction in monolayer MoS_2_ films that are suspended over holes with a size in the micrometer range after they are ruptured. This study proved that vacancy formation in MoS_2_ that form line defects is origin of crack formation to increase fracture toughness.^[^ ^115^ ^]^ Dislocation formation is limited to the crack tips due to the requirement of Mo and S atom loss for the creation of edge dislocation cores, which is commonly not observed in MoS_2_. Therefore, holes, cracks, and the termination of domains or grains provide useful avenues for studying the edge structures of 2D TMDs. Due to the presence of two elements in TMDs, their edge behavior is different from that of graphene, which only contains carbon. Armchair edges are not commonly found in TMDs, whereas zigzag edges are dominant.^[^ ^85^, ^116^, ^117^ ^]^ The zigzag edges have two possible terminations—chalcogen and metal—which show diﬀerent thermodynamic stability. Using a high‐temperature heating holder, a recent study revealed that MoS_2_ demonstrates exceptionally smooth zigzag edges when heated above 500 °C. The edge shows two distinct contrast patterns which correspond to the different zigzag terminations, chalcogen and metal. At room temperature, the edges of chemically vapor‐grown MoS_2_ samples typically terminate along the zigzag direction with terraced step edges. High‐temperature ADF‐STEM imaging of the edges showed that a sulfur‐poor environment in MoS_2_ triggers the reconstruction of S‐terminated zigzag edges to become double Mo‐terminated.

### 5.5 Diffusion and Rearrangement of Adatoms

Most of the ejected atoms at the surface of 2D materials do not leave the specimen completely. These atoms have weak bond with the surface, which make them easily diffuse along the surface because of the three‐order lowered activation energy compared to adsorption energy.^[^ ^118^ ^]^ Romdhane et al. observed the fact that these adatoms are aggregated and rearranged themselves at the defect sites and on the surface, helping the growth of Cu_2_S in the TEM environment.^[^ ^119^ ^]^ The self‐repair of Bi_2_Te_3_ and MoS_2_ under 300 kV electron irradiation has been recently reported by Shen et al. Bi, Te, Mo, and S atoms ejected from the lattice were diffused into nanopores, which provided source for healing.^[^ ^120^ ^]^ Observations made by in situ STEM have shown that adatoms preferentially occupy sites with more surrounding atom columns. This is because these sites offer a greater binding energy, which is due to the additional bond formation that occurs. Electron irradiation can be the way to fabricate 2D materials. Xu et al. demonstrated that the rates of atom ejection and reconstruction may vary depending on the electron beam intensity,^[^ ^121^ ^]^ so optimizing electron beam intensity is crucial to carry out desirable fabrication. Quang et al. showed that if enough incoming energy is applied to induce structural reconstruction, novel 2D or quasi‐2D structures can also be formed from clusters on the specimen surface. Free‐standing graphene like ZnO monolayer is formed either on graphene substrates or inside graphene nanopores.^[^ ^122^ ^]^ Yin et al. observed similar phenomena; formation of single‐atom thick copper oxide layers on graphene substrate and nanopores.^[^ ^123^ ^]^ Although the results of these studies are based on microscopy observations, they demonstrate the potential of using graphene as a substrate for growing 2D membranes and offer a new approach for depositing 2D materials onto graphene. The mechanism of migration of adatoms is studied by Liu et al. Single heteroatoms located at the edge of graphene can act as catalytic sites for the deposition of adatoms that diffuse to the edge in the presence of foreign atoms.^[^ ^124^ ^]^ Ta et al. reported that Cr atoms also show in situ catalytic growth in the transmission electron microscope.^[^ ^125^ ^]^ Under electron beam, Cr atoms move randomly along the edge and settle on single and double graphene vacancies. This movement facilitates the insertion of carbon atoms between the initial and final positions of Cr atoms, leading to the formation of a new hexagonal lattice in graphene. In contrast, J. Zhao's study revealed that Fe atoms exhibit distinct behavior in a similar environment. They diffuse along a graphene edge, causing the removal or addition of C atoms and thereby etching or growing graphene.^[^ ^126^ ^]^

### 5.6 Electron Beam‐Induced Crystallization

The use of electron beam‐induced irradiation has been found to induce the crystallization of 2D or quasi‐2D crystals. Borrnert et al. reported that when amorphous carbon is under electron beam irradiation, it is heated and causes graphitization.^[^ ^127^ ^]^ The growth of graphene can be modulated through optical tuning in TEM when amorphous carbon is deposited on graphene or h‐BN membranes. When the amorphous carbon gets focused electron beam irradiation in STEM mode, it causes holes in the graphene or BN support. When amorphous carbon undergoes parallel electron beam irradiation, it undergoes graphitization and subsequently experiences layer‐by‐layer growth.^[^ ^127^ ^]^ Amorphous MoS_2_ can also be crystallized under electron beam, which is studied by Bayer et al.^[^ ^128^ ^]^ The thermal energy transferred from the electron beam, which is similar to ex situ heating treatment associates to overcome kinetic barriers to crystallization.^[^ ^129^, ^130^ ^]^ Studies have shown that the electron beam can effectively trigger nucleation and growth of 2D materials, even with small crystalline or amorphous 2D materials. From this point of view, the in situ TEM study of formation of 2D crystals is applicable to other experimental conditions outside the microscopy.

## 6 Data‐Driven and Computational Analysis of 2D Materials

The AC‐STEM analysis has provided atomic‐scale insights for understanding atomic structure of materials and structure–property relations. Building a reasonable structure–property relationship in a complex system is challenging due to errors associated with human interpretation in Z‐contrast STEM image analysis, as well as the relatively low throughput and accuracy of the analysis. In 2D materials, imaging at low‐voltage is inevitable to avoid knock‐on damage, so STEM images are acquired with low signal‐to‐noise ratio (SNR), causing analysis with low accuracy.

**Figure 11:**
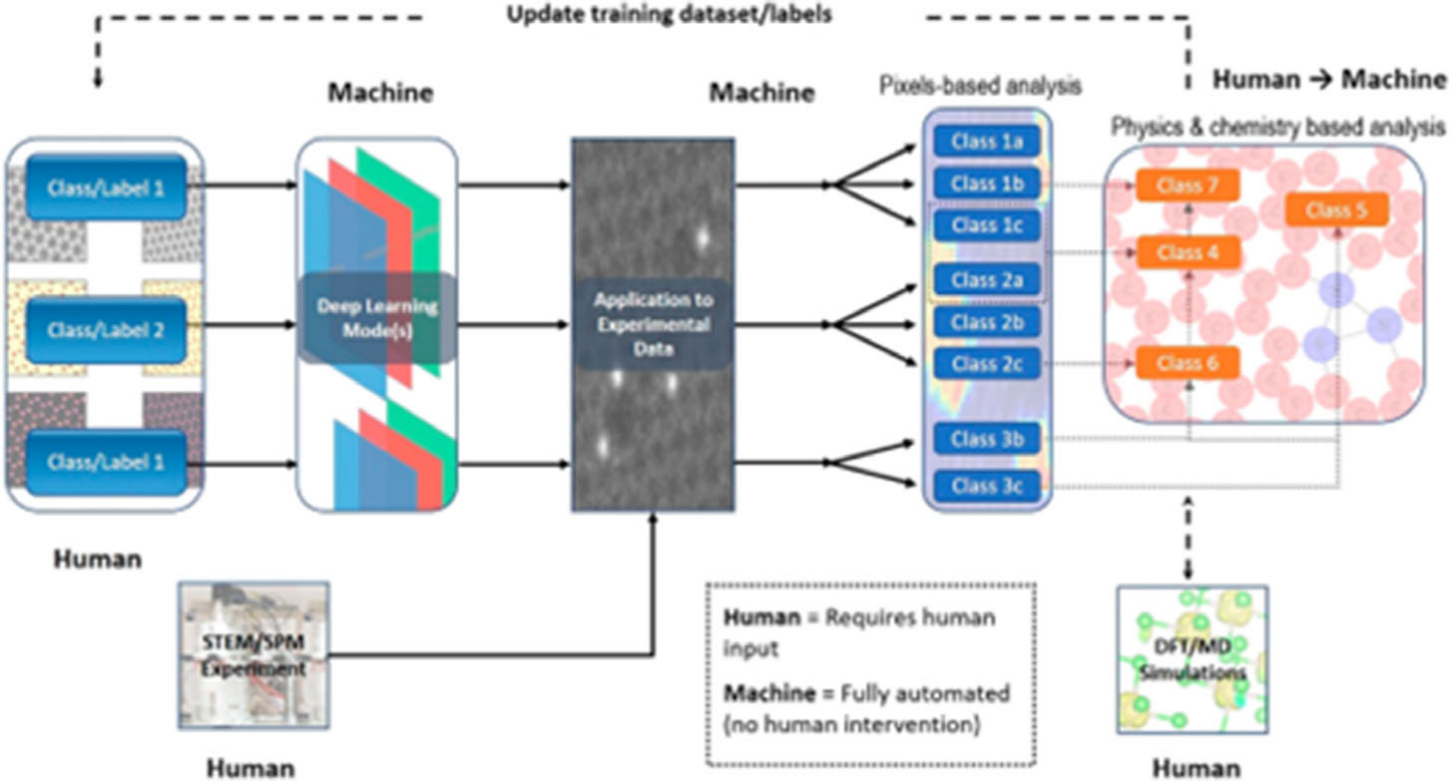
Schematics of a “weakly supervised” method for identifying lattice configurations and defects in experimental data. The process involves both human and machine input, which is indicated by the labels “Human” and “Machine.” The details of the process are explained in the accompanying text. Adapted with permission.^[^ ^131^ ^]^ Copyright 2017, American Chemical Society.

In recent decades, automated analysis such as machine learning has been developed rapidly and it is opening new era of STEM analysis. The combination of the previously mentioned advancements, such as clustering, classification, and reinforcement learning, has led to the expansion of machine and deep learning into multiple scientific domains. **Figure** **11**'s right panel highlights some of the significant milestones in this area.^[^ ^131^ ^]^

The advent of machine learning in interpreting atomic structures and defects in STEM images, especially of 2D materials, has emerged as a new platform for image analysis with high accuracy with the qualitative manner. Automated analysis has the potential to reveal insights and correlations in data that may have been overlooked in traditional manual analysis methods. Numerous research works have been conducted to develop automated algorithms for identifying structures from different microscopy data, including but not limited to identifying vacancies in 2D materials,^[^ ^132^, ^133^ ^]^ segmenting phases in catalysts,^[^ ^133^, ^134^ ^]^ crystallography determination by deep learning,^[^ ^135^ ^]^ and manifold learning of electron ptychography data.^[^ ^136^ ^]^ Automated platforms with high efficiency enable real‐time mass defect analysis and structural identification, making computational electron microscopy more feasible than ever before. The ultimate aim of the advancement in instrumentation and quantitative techniques is to uncover more comprehensive relationships between the structure and properties of materials, which can facilitate the design of materials with desirable functionalities.

### 6.1 An Integrated Approach to Automate the Analysis of Atomic Structures

Structural analysis in atomic resolution STEM imaging in quantitative manner can be divided into three main stages: detection of atomic columns, information representation (of structural features), and identifying atomic structures. During the process of atomic column detection, various techniques such as model‐based methods,^[^ ^137^, ^138^, ^139^, ^140^, ^141^, ^142^, ^143^ ^]^ moment‐based methods,^[^ ^144^ ^]^ scale‐space methods,^[^ ^145^, ^146^ ^]^ etc. are employed to determine the precise position of each atomic column. The positions of atomic columns are detected and referred to as either feature points or key points. Following this, a stack of local patches is extracted and forms a 3D matrix when a window size *m* is applied during the information representation stage. To mitigate the challenge posed by high dimensionality and complex computation, one option is to flatten each patch and convert it into a feature vector with a single dimension. The complete collection of feature vectors can be merged or arranged into a matrix of features. In the third stage, the feature matrix is utilized as an initial step for applying unsupervised and supervised machine learning methods to detect all potential atomic structures. To summarize, the three stages aim to achieve the following: first, to detect the positions of atomic columns for specific applications; second, to develop a method to transform the local patch into a descriptive feature vector; and third, to choose an appropriate identification scheme.

### 6.2 Detection of Atomic Columns

This section will discuss three prevalent methods used for detecting and identifying atomic columns. The discussion will cover their fundamental principles, mathematical formulations, as well as their advantages and disadvantages. Furthermore, this section will examine the practical aspects of implementing atomic column detection algorithms and their relevance to atomic structural identification. Additionally, a suggested approach for developing new identification algorithms will be provided.

#### 6.2.1 Model‐Based Fitting Method

In the model‐based fitting method, intensity of an atomic resolution STEM image can be fitted as a superposition of 2D Gaussian functions.^[^ ^137^, ^138^, ^139^, ^140^, ^141^, ^142^, ^143^ ^]^ This method's main benefit is that it can achieve subpixel precision detection of atomic columns by optimizing the fitted arguments. This ability is particularly useful for strain field mapping^[^ ^147^ ^]^ and measuring bonding angles.^[^ ^139^ ^]^ However, the accuracy of position identification using this model may be negatively affected by background intensity caused by surface contaminants, lens aberrations, or scanning noise. Another drawback of this algorithm is that it is computationally intensive, even if all parameters are effectively initialized.

#### 6.2.2 Moment Method

The moment method is significantly faster than model‐based Gaussian fitting.^[^ ^148^ ^]^ By starting with a group of initial positions, the central point within the surrounding area of each point of concern can be determined through iterative calculations. Because this method is sensitive to noise, the moment method is useful in strain analysis due to the reduced accuracy. The size of the region for centroid calculation also determines the positions. To make the algorithm to be proceeded, initial positions are required for both the moment method and the model‐based method. However, it can be challenging to determine the number of atomic columns using the moment method in multiphase systems.^[^ ^149^ ^]^ This is because multiple sets of basis vectors need to be specified in the preprocessing stage, which adds to the complexity. Recently, Fatermans has proposed a solution to this problem by integrating Bayesian statistics to achieve the most favorable atomic column position and number in low SNR STEM images.^[^ ^150^ ^]^ Still, it is not optimized for real‐time structure analysis due to complex nature of this algorithm. From this standpoint, methods characterized by reduced time complexity and not requiring the optimization of an initial set of positions can be viewed as potential options for enhancing the algorithms.

#### 6.2.3 Scale‐Space Method

Methods based on scale‐space, such as Laplacian of Gaussians (LoG),^[^ ^145^ ^]^ difference of Gaussians (DoG),^[^ ^146^ ^]^ and Hessian of Gaussians (HoG), have the advantage of not requiring the initial positions to be provided because they can automatically search for the atomic columns algorithmically. These techniques are classified as blob detection algorithms that are adaptable for various types of high‐resolution microscopy images, including STEM, AFM, and STM, among others. This is because atomic columns resemble the concept of blobs in scale‐space theory, and thus these methods are suitable for a wide range of atomically resolved microscopy images. According to Marsh, the fundamental idea in scale‐space methods is convolving the input image with multiple LoG kernels that have different scale parameters “*t*.”^[^ ^150^ ^]^ The input image, which contains information about position and scale, is transformed into a series of smoothed images that have been convolved with kernels of varying scales. Algorithms for detecting blobs, such as the maximum filter method, are utilized within the image stack to identify the location, magnitude, and curvature data of the original input image. The subpixel refinement process is essential for applications in strain mapping analysis and octahedral tilt measurement. According to Radar, the distinctive characteristic of scale‐space methods is their utilization of fast Fourier transformation (FFT) to accelerate the computation, which results in a log‐linear time complexity.^[^ ^151^ ^]^ Once the initializaion of position is eliminated, atomic columns are automatically detected without tedious preprocess, which simplifies the overall process of the automated analysis.

### 6.3 Structure Identification via Machine Learning

Machine learning is very powerful tool when STEM images with massive atomic scale have to be analyzed for the atomic structure identification problem in a quantitative manner. Many structural identification schemes that have been applied in AC‐STEM images use this algorithm. In general, algorithms for structure identification and analysis are divided into two parts: supervised learning and unsupervised learning. Cluster analysis is a technique in unsupervised learning that involves dividing a dataset into distinct groups based on the similarities between the data points. In solving structure identification problems, the crucial task is to automatically group together feature vectors of the same type. Cluster analysis is a crucial task in the automatic grouping of similar feature vectors in structure identification problems. This involves different algorithms, including *K*‐means,^[^ ^152^ ^]^ hierarchical clustering, and density‐based spatial clustering of applications with noise (DBSCAN),^[^ ^153^, ^154^, ^155^ ^]^ which aim to divide mixed datasets into subgroups based on similarities. The success of each approach relies on the quality of the feature vectors extracted in the second stage. *K*‐means clustering aims to group a set of feature vectors into *k* groups with equal variances, as suggested by its name. The number *k* is an important input parameter for *K*‐means, indicating that some prior knowledge about feature vectors must be available beforehand. When it comes to determining atomic structures, it is essential to have prior knowledge, such as crystal structure and the number of polymorphs, in order to select *k* appropriately. *K*‐means is not effective in analyzing defects in 2D materials. This is because when the number of point defects or dopants is low, there is a significant difference in concentration between the defects and the original lattice, resulting in uneven‐sized clusters in *K*‐means that produce an invalid model. *K*‐means may not be an effective approach for analyzing low concentrations of point defects or dopants in 2D materials due to the substantial difference in concentration between defects and pristine lattices, which causes uneven‐sized clusters in *K*‐means and renders the model invalid. Hierarchical clustering and DBSCAN are more suitable for cases where clusters of various shapes and sizes are present. DBSCAN uses density estimation of feature vectors and can handle clusters of different shapes and sizes by specifying the maximum distance between feature vectors that can be considered in the same neighborhood with the input parameter *ε*. In order to address the limitation of specifying only one value for *ε* in DBSCAN, the OPTICS algorithm has been introduced as a more flexible approach that allows for multiple ranges of *ε* values. The parallel HDBSCAN algorithm is beneficial for quick structure identification when dealing with extensive datasets. This algorithm enables the extraction of clusters with varying densities. Density‐based methods may face the problem of having some feature vectors that cannot be labeled, resulting in unclear cluster structures. Hierarchical clustering methods are utilized to achieve a more complete understanding of cluster structures, producing a dendrogram that demonstrates similarities among feature vectors and can be used to determine the quantity of clusters. The best clustering algorithm for feature vectors with an unknown topological structure is not determined by any specific criterion. Manifold learning is considered as an empirical routine that connects the first stage and the second stage for analyzing structures in atomically resolved microscopy images. In particular, after reducing the dimensionality of image patches that encode structural information, compressed feature vectors are commonly obtained to facilitate interpretation. These descriptors are then input to the manifold learning model to enhance separability, which can improve the clustering process. This dual representation via descriptors and manifold learning can simultaneously achieve interpretation and separation.

### 6.4 Deep Learning

Recently, deep learning techniques, such as recurrent neural networks, transformer, and convolutional neural network, have strongly influenced in the fields of automated analysis of images and even material informatics. Deep learning is classified into supervised, unsupervised, semisupervised, or reinforced. Supervised deep learning algorithms learn a nonlinear function that connects the input atomic structure to an existing structure, whereas unsupervised learning learns a linear function that indirectly relates the input to the structure. If a significant amount of training data is provided, the model is capable of identifying atomic structures. Ziatdinov et al.^[^ ^156^ ^]^ have recently created weakly supervised deep neural networks that can identify atomic configurations of silicon atoms on graphene, along with their symmetry dynamics when exposed to electron beam irradiation, by extracting data from atomically resolved images. This technique has the potential to be expanded to extract and analyze information from experimental data on a larger scale. A similar deep learning model has also been established by Ziatdinov et al.^[^ ^131^ ^]^ to find point defect dynamics on monolayer WS_2_ lattices in sequential STEM images. Thousands of experimental STEM images are extracted for training data. The potential of deep learning algorithms to generalize and analyze microscopic data is strongly dependent on the training data set extracted from raw STEM images. A common approach for acquiring sufficient training data for analyzing STEM images involves generating simulated images using various augmentation techniques. The creation of simulated images depends on existing information. For instance, when detecting point defects, Ziatdinov collected simulated images of 2D materials with defects and dopants, and generalized the dopants with higher atomic number and vacancies.^[^ ^131^ ^]^ Analyzing point defects in STEM images using deep learning is a difficult task because of the significant disparity between the number of available training data and the variety of defect types. In addition, the deep learning model becomes less effective with complicated background information in STEM images which is from prevalent surface contamination on 2D materials or scanning noise when obtaining STEM images. In order to make deep learning model successful, minimizing noise and surface contamination is essential. Only improving deep learning model cannot overcome this issue.

### 6.5 4D STEM Analysis: A Brand‐New Approach

4D STEM analysis is a new emerged approach to analyze materials, and this method is especially applicable to 2D materials. In this subsection, we introduce principle of 4D STEM briefly and discuss what 4D‐STEM has brought to the field. 4D STEM record a pixelated, angle‐resolved 2D convergent beam electron diffraction (CBED) pattern for every position in the 2D STEM raster over the sample, providing a 4D dataset. Compared to conventional AC‐STEM imaging, the key advantage of 4D STEM is a single scanning can obtain a 4D dataset that contains all possible STEM images of the specimen. With 4D datasets, virtual apertures can be inserted by extracting only the desired diffraction points, which enables virtual dark field (DF), HAADF, annular bright field (field), iDPC imaging.

4D STEM also enables the examination of electric and magnetic field structures at the atomic scale, offering valuable information for analyzing local chemistry defects,^[^ ^157^, ^158^, ^159^, ^160^ ^]^ mapping distortions in the lattice and spacing between layers in 2D materials,^[^ ^150^ ^]^ and even visualizing anionic electrons.^[^ ^161^ ^]^

In the case of 2D materials, the 4D stem technique complemented the existing STEM method's limit to clearly reveal the structure due to resolution problems, especially in twisted bilayers or trilayers. Zachmann et al. applied interferometric 4D STEM to describe twisted bilayer and trilayer graphene picometer‐scale distortion which STEM and conventional STEM imaging and STEM technique could not observe.^[^ ^162^ ^]^ Roccapriore et al. combined deep‐kernel learning with 4D STEM to analyze twisted bilayer graphene and mesoscale‐ordered patterns in the MnPS_3_ in precise.^[^ ^161^ ^]^ Mehta et al. used virtual dark field imaging in combination with unsupervised learning to elucidate stacking order of epitaxial bilayer MX_2_ layers (M: Mo, W; X: S, Se).^[^ ^163^ ^]^ Xu et al. utilized 4D STEM technique to verify Moire structure of CrI_3_. Due to the unique structure, ferromagnetic and antiferromagnetic states coexist in the CrI_3_.^[^ ^164^ ^]^ Strain field mapping of twisted bilayer graphene using atomic displacement fields which is measured by 4D STEM Bragg interferometry was carried out by Kazmierczak et al.^[^ ^156^ ^]^

Using the 4D STEM technique mapping physical parameters (e.g., electric field, polarization) of the 2D materials was carried out. Xu et al. measured polarization field of In_2_Se_3_ by analyzing asymmetric intensity of CBED pattern, which can be obtained by 4D STEM.^[^ ^165^ ^]^ Similarly, electrostatic field mapping of MoS_2_ and WS_2_ was carried out by Fang et al., showing that defective areas can act as conducting channels.^[^ ^166^ ^]^

4D STEM technique is also used to retrieve phase information, which is called ptychography. Conventional AC‐ADF or HAADF STEM is beneficial to detect heavy elements but shows disadvantage with observing light elements. With ptychography, not only heavy elements but also light elements such as carbon, nitrogen can be visualized in superior resolution. Jiang et al. recovered phase information of MoS_2_ and therefore, spatial resolution has been improved beyond numerical aperture.^[^ ^167^ ^]^ Wen et al. used ptychographic phases reconstructions to simultaneously observe heave and light elements from electron beam‐induced carbon contaminated MoS_2._^[^ ^168^ ^]^ Differential phase contrast imaging employs quadrant segmentation of 2D pixelated direct electron detector data, which not only demonstrates qualitative agreement with ptychographic phase reconstructions in terms or resolution and contrast but also provides the added benefit of real‐time display capability.

The integration of machine/deep learning techniques with the 4D STEM technique presents a solution to the limitations of analysis encountered in conventional AC‐STEM, including resolution and light element detection. Moreover, it proves highly beneficial for extracting physical properties of materials, such as strain, electric field, and polarization mapping. Future research efforts should explore the potential of 4D STEM in extracting not only structural information but also diverse physical and chemical information. To ensure higher accuracy in extracting such information, the development of techniques with enhanced precision becomes imperative.

## 7 Conclusions and Outlook

Investigating structure–property relationship of 2D materials is a key for achieving their controlled properties and applications. To give feedback to synthesis as well as tailoring properties of 2D materials, it is significantly important to observe atomic structures, defects, and growth mechanisms. For this, STEM imaging is a powerful tool to observe atomic arrangements due to superior spatial resolution. Furthermore, STEM imaging allows us to discover atom dynamics due to interaction between electron beam irradiation and 2D specimens. This review article summarizes recent research that utilizes STEM at the atomic resolution, encompassing topics such as atomic structure, defects, in situ observation, and computational analysis including automated analysis, machine/deep learning, and 4D‐STEM. Studies of this kind have provided detailed explanations and important understandings of the atomic structure of 2D materials. **Table** **1** shows the summary of this review article. However, there are still several challenges, opportunities, and future directions.

**Table 1:** Summary of observation of 2D materials using TEM/STEM

Material	Objective (goal)	Results	References
Graphene	Finding optical condition of observing graphene	STEM observation of graphene under 100 kV	[17]
h‐BN	Studying atomic structure of h‐BN	Edge structures and defects as point defects, dislocations, and grain boundaries	[12, 13, 16, 18, 19, 20]
h‐BN	Studying atomic structure of h‐BN	Stacking sequence	[21]
h‐BN	Mapping electric fields	Enhanced electric fields around defects of h‐BN	[26]
MoS_2_	Observation of MoS_2_ polymorphs	Distinguishable atomic arrangements of 1H, 1T, 2H, and 3R‐MoS_2_	[35, 36]
BP	Cross‐sectional observation of BP thin films	Armchair and zigzag atomic arrangement of BP	[44, 45, 46, 47]
BP	Visualization of structural anisotropy	Different atomic arrangements in 3 zone axis	[48]
MXene	Synthesis of ordered MXenes	Mo_2_Ti_2_C_2_ and Mo_2_TiAlC_2_	[54]
MXene	Atomic investigations of single MXene	Point defects of Ti_3_C_2_Ti_*x*_ which cause formation of TiO_2_ on surface	[56, 57, 58]
MoS_2_	Investigation of intrinsic structural defects	Vacancies, edges and grain boundaries of MoS_2_	[61]
MoS_2_	Investigation of intrinsic structural defects	Nanopores in MoS_2_	[62]
MoS_2_	Investigation of atomic reconstruction of line defects	Line defects of MoS_2_	[63]
Mo_1−*x*_W_*x*_S_2_	Confirmation of atomic distribution	Odd distribution of Mo and W atoms	[64, 65, 66, 67]
MoS_2(1–*x*)_Se_2*x*_	Confirmation of atomic distribution	Odd distribution of S and Se atoms	[68, 69]
WS_2(1–*x*)_Se_2*x*_	Figuring out substitutional site	Random distribution of S and Se atoms	[70]
MX_2*x*_Te_2(1–*x*)_	Figuring out substitutional site	Phase‐dependent ordering (1T′ anisotropic, 2H isotropic)	[71]
Re‐doped MoS_2_	Stability under electron irradiation	Stable Re atoms at Mo sites	[73]
Re‐doped MoS_2_	Figuring out substitutional site		[74]
Nb‐doped WS_2_	Structural characterization	Nb dopants on W site	[75]
MoS_2_	Effects of doping on structure of MoS_2_	Dopant did not change the atomic structure of MoS_2_	[76]
Pt‐doped MoS_2_	Observing dynamics of Pt dopants	Positional preference of Pt atoms on MoS_2_	[77]
Co‐doped MoS_2_	Figuring out site of Co atoms	Co atoms prefer S vacancy sites	[78]
MoS_2_	Observation of vacancy migration	Preferred pathways of vacancy migration	[79]
MoS_2_ with Pt	Observation of epitaxial grown Pt	In situ growth of Pt nanocrystal on MoS_2_	[80]
MoS_2_ with CuCl_2_	Observation of in situ growth	Epitaxial growth of CuCl_2_ on MoS_2_	[81]
MoSe_2_	Investigation of defects on conductivity	Line defects providing conducting channel	[82]
MoS_2_	Observation of defects on MoS_2_	Grain boundary of MoS_2_	[83]
MoS_2_	Figuring out atomic structure of grain boundary	5 and 7 rings on grain boundary of MoS_2_	[84, 85]
MoS_2_	Observation of defects on MoS_2_	Antiphase boundary	[86, 87]
BN/graphene/WS_2_	Cross‐sectional observation	Vertical heterostructure	[88]
WS_2_/MoS_2_	Observation of edge structure of interface	Clear and zigzag edges at the interface	[90]
WSe_2_/SnS_2_	Plan view observation	Moire lattice	[91]
GaSe/MoSe_2_	Plan view observation	Moire lattice	[92]
h‐BN/WS_2_/h‐BN	Cross‐sectional observation	Atomic defects at the interface	[93]
WS_2_–WSe_2_ and MoS_2_–MoSe_2_	Plan view observation	2D lateral heterostructures without overlap	[94]
WSe_2_/MoSe_2_	Plan view observation	Heterostructure with clear interface	[95]
Re‐doped MoS_2_	In situ observation	2H to 1T phase transition of Re‐doped MoS_2_	[101]
MoTe_2_	In situ observation	Point defects transformed to line defects under electron beam irradiation	[105]
MoTe_2_	In situ observation	Formation of domain boundaries under heat	[106]
Pd_2_Se_3_	In situ observation	Melding of Pd_2_Se_3_ under electron beam irradiation	[108]
WSe_2_	In situ observation	Formation of threefold rotational defects under electron beam irradiation	[112]
CrO_2_ on graphene	In situ observation	Growth of CrO_2_ nanocrystal on graphene	[113]
SnS_2_	In situ observation	Transformation of SnS_2_ to SnS	[114]
MoS_2_ and graphene	In situ observation	Graphene protecting damage from electron beam irradiation	[116]
NbSe_2_ and graphene	In situ observation	Graphene preserving NbSe_2_	[117]
MoS_2_	In situ observation	Crack tip and stress corrosion of MoS_2_	[85]
Cu_2_S	In situ observation	In situ formation of Cu_2_S nanocrystal	[119]
MoS_2_, Bi_2_Te_3_	In situ observation	In situ repair of nanopores	[120]
Graphene	In situ observation	Deposition or sculpting depending on acceleration voltage of TEM	[121]
Graphene and ZnO	In situ observation	ZnO deposited on graphene	[122]
Graphene and CuO	In situ observation	CuO deposited on graphene	[123]
Graphene	In situ observation	Si atom mediated in situ growth of graphene	[124]
Graphene	In situ observation	Catalytic growth of graphene with Cr atom	[125]
Graphene	In situ observation	Catalytic growth of graphene with Fe atom	[126]
Graphene with amorphous C	In situ observation	Deposition or breaking of graphene depending on optical condition	[127]
MoS_2_	In situ observation	Crystallization of MoS_2_ under electron beam irradiation	[128]

Direct imaging of defects in 2D materials is still the main research directions for the future. It is expected that research will be conducted in two directions: the development of analytical techniques and the identification of phenomena through imaging. Development of quantitative defect analysis will bring more information for defect analysis. Researchers will be able to extract quantitative information about defect size, shape, strain fields, and chemical composition. This detailed analysis will enhance our understanding of defect formation, evolution, and their influence on material properties. Aberration‐corrected STEM imaging combined with advanced machine learning algorithms will enable automated defect classification. By training machine learning models on large datasets of labeled defect images, it will be possible to identify and classify different types of defects in 2D materials more efficiently. This will accelerate defect analysis and provide valuable statistics about defect distributions and types.

In terms of heterostructure, AC‐STEM imaging shows many possibilities. There are many electromagnetic properties that have not yet been discovered in heterojunctions. Finding the exact arrangement of atoms will continue to be essential for materials scientists who want to understand the structure–property relationship. AC‐STEM imaging can provide valuable insights into the local electromagnetic responses of heterostructures. By combining AC‐STEM with techniques such as EELS or cathodoluminescence (CL) spectroscopy, it becomes possible to map the plasmonic, excitonic, or photonic properties of individual layers within the heterostructure. This can help identify localized excitations, energy transfer processes, and how the electromagnetic properties of one layer can influence adjacent layers. Heterostructures can exhibit fascinating surface plasmon resonances, which are collective electron oscillations resulting in enhanced electromagnetic fields at the nanoscale. AC‐STEM imaging combined with low‐loss EELS can provide spatially resolved information about these plasmonic modes and their coupling with specific layers or interfaces within the heterostructure. Understanding and engineering such plasmonic modes can lead to advancements in nanophotonics, sensing, and light–matter interactions.

By combining different 2D materials in heterostructures, hybrid systems can be created with enhanced or novel electromagnetic properties. AC‐STEM imaging can play a crucial role in characterizing and understanding the interfaces, interlayer coupling, and structural defects within these heterostructures, which directly influence their electromagnetic response.

In terms of real‐time observation, AC‐STEM study shows a lot of potential for advancement. In situ observation enables the study of dynamic processes in 2D materials, such as growth, phase transitions, chemical reactions, and mechanical deformations. By imaging the materials in real time during these processes, researchers can gain insights into the underlying mechanisms, kinetics, and structural changes that occur at the atomic scale. This knowledge is crucial for tailoring the synthesis and processing of 2D materials for specific applications. In order to observe the growth process of the 2D material in real time, as well as to see the interaction between the electron beam and the 2D material, manipulating the environment inside TEM is needed. Parameters such as temperature, pressure, humidity, and gas composition can be precisely adjusted, enabling the investigation of how these external factors influence the properties and behavior of 2D materials. Future advancements will focus on expanding the range of controllable environmental conditions and integrating multiple in situ techniques for comprehensive characterization. In situ AC‐STEM study can also be combined with electrical and mechanical measurements to study the response of 2D materials under applied fields or mechanical stress. Techniques such as in situ electrical probing and nanoindentation allow researchers to investigate the electrical conductivity, charge transport, and mechanical properties of 2D materials under real‐time conditions. These studies are crucial for developing high‐performance electronic and optoelectronic devices based on 2D materials.

In addition, to elucidate the growth mechanism of 2D material films, investigating atomic and electronic structure of film/substrate of 2D material heterointerface is important. Although van Der Waals interaction is dominant between layers of 2D materials, its growth is depending on the template. Studying atomic dynamics such as domain nucleation and ferroelectric switching guide us to control and optimize the ferroelectric properties of 2D materials which have potential to be used as memory and neuromorphic devices.

Finally, the development of data‐driven analysis can overcome the analysis limitations of existing 2D materials, and the use of this analysis technique is expected to provide much higher quality information in understanding 2D materials. In this respect, computational analysis is useful for AC‐STEM imaging of 2D materials. In terms of automated analysis, the future perspective involves refining and expanding automated analysis tools to handle increasingly complex datasets, improve accuracy, and enhance the integration of multiple characterization techniques. Developing machine/deep learning analysis technique should be the direction of the research, which includes developing learning architectures to handle complex AC‐STEM data, improving the accuracy and efficiency of defect identification and classification, and enabling real‐time analysis for in situ observation.

More importantly, 4D STEM technique should be further developed along with advances in machine learning and deep learning. In terms of material science, it is necessary to continuously consider what kind of physical and chemical data should be extracted through the 4D system, and in terms of analysis techniques, a computer‐based technology that can extract these physical and chemical data using 4D datasets is required. Developments of 4D STEM technique will focus on improving data acquisition speed and sensitivity, as well as advancing data analysis techniques. This includes developing algorithms for rapid and efficient reconstruction of high‐quality images from the 4D datasets, improving sensitivity for mapping subtle variations in composition and strain, and integrating machine learning approaches for automated analysis and interpretation of complex data.

## Conflict of Interest

The authors declare no conflict of interest.
